# Structural basis of heme scavenging by the ChtA and HtaA hemophores in *Corynebacterium diphtheriae*

**DOI:** 10.1016/j.jbc.2025.110633

**Published:** 2025-08-26

**Authors:** Jordan Ford, Andrew K. Goring, Yuri Lee, Megan Chen, Brendan J. Mahoney, Michael R. Sawaya, Hannah S. Shafaat, Joseph A. Loo, Robert T. Clubb

**Affiliations:** 1 Department of Chemistry and Biochemistry, University of California, Los Angeles, Los Angeles, California, USA; 2 UCLA-DOE Institute of Genomics and Proteomics, University of California, Los Angeles, Los Angeles, California, USA; 3 Molecular Biology Institute, University of California, Los Angeles, Los Angeles, California, USA

**Keywords:** bacterial pathogenesis, conserved region domain, X-ray crystallography, resonance Raman spectroscopy, electron paramagnetic resonance, native mass spectrometry, hemophore, heme, iron, *Corynebacterium diphtheriae*, isothermal titration calorimetry

## Abstract

*Corynebacterium diphtheriae* causes diphtheria, a potentially fatal infectious disease that damages tissues in the upper respiratory tract. In order to proliferate, this pathogen acquires the essential nutrient iron from heme (iron-protoporphyrin IX) primarily found in human hemoglobin (Hb). *C. diphtheriae* secretes ChtA and HtaA hemophore proteins that bind ferric heme (hemin) *via* conserved region (CR) domains. Here, we demonstrate that their CR domains scavenge hemin after it is spontaneously released from Hb, and define the structural basis of hemin binding to ChtA and the N-terminal CR domain from HtaA by determining X-ray crystal structures of their protein-hemin complexes. Resonance Raman and electron paramagnetic resonance experiments demonstrate that the CR domains from ChtA and HtaA engage in pentacoordinate hemin binding through a conserved iron-tyrosyl linkage, though variations in their hemin pockets alter the way they stabilize the axial tyrosine and mask hemin’s metal. The importance of these interactions is probed using isothermal titration calorimetry experiments, which represent the first quantitative assessment of CR-hemin affinity and reveal that ChtA binds hemin *via* an enthalpically driven process. Hemin partitioning experiments using native mass spectrometry demonstrate that the cohort of CR domains within *C. diphtheriae*’s hemin-uptake system have dissociation constants for hemin between 0.8 and 22 nM, raising the possibility that affinity differences contribute to the directional flow of hemin into the cell. Collectively, the results of this work provide insight into how *C. diphtheriae* and other pathogenic and commensal corynebacterium species utilize CR domains to scavenge iron rich hemin from their environment.

Bacterial pathogens require iron in order to meet their metabolic needs, as this metal is a vital cofactor for microbial enzymes that mediate key cellular processes such as DNA replication, electron transport, and amino acid synthesis (1, 2). During infections, many pathogens forage iron from heme (iron-protoporphyrin IX), which contains >75% of the human body's total iron content (3, 4, 5). The vast majority of heme is located within human hemoglobin (Hb), which is released from red blood cells by the action of bacterial cytolysins or as a result of spontaneous lysis of senescent cells (6). Once in the extracellular environment, Hb-bound heme is rapidly oxidized to its ferric state (called hemin) to produce methemoglobin (metHb) (7, 8). To limit iron access to invading pathogens and to avoid host tissue damage caused by hemin-produced reactive oxygen species (9), both metHb and free hemin are rapidly removed from the blood. MetHb is bound by the abundant human plasma protein haptoglobin (Hp) and subsequently cleared by macrophages *via* CD163-mediated endocytosis (10, 11), while free hemin is bound by hemopexin and subsequently cleared from the blood by hepatocytes and macrophages (12, 13, 14). In order to gain access to this limited resource, many bacterial pathogens employ uptake systems that scavenge and import host hemin that is released from Hb (15). A greater understanding of these systems could facilitate the development of therapeutically useful heme-uptake inhibitors that would limit infections by starving bacteria of iron.

*Corynebacterium diphtheriae* is the causative agent of the life-threatening respiratory disease diphtheria (16). Epidemic isolates encode five extracellular proteins that bind hemin *via* conserved region (CR) domains: ChtA, ChtB, ChtC, HtaA, and HtaB (17) (Fig. 1). Genes encoding these proteins and other components of the hemin uptake system are located within five genetic loci (*hmu*, *chtA-chtB*, *cirA-chtC*, *hbpA*, and *hmuO*) (18, 19, 20). Their expression is upregulated in iron-depleted conditions by the action of the diphtheria toxin repressor, which also controls the expression of other virulence-related proteins, including that of the diphtheria toxin (21). Strains harboring deletions in heme uptake system components are impaired in their ability to use Hb or the Hb:Hp complex as an iron source (22). Based on their primary sequences, the surface-attached and secreted proteins in the heme uptake systems contain a single CR domain, with the exception of HtaA, which contains two of these modules (hereafter referred to as CR1 and CR2, with CR1 being N terminal to CR2). They also contain a C-terminal nonpolar amino acid segment that presumably forms a transmembrane helix that tethers each protein to the extracellular plasma membrane. Recent proteomics studies revealed that the ChtA, ChtC, and HtaA proteins are exposed on the cell surface, whereas the ChtB and HtaB proteins are buried within the cell envelope (23). Interestingly, significant amounts of ChtA and HtaA are also secreted, and thus presumably moonlight as hemophores that scavenge hemin from the environment (23). Collectively, the CR-containing hemoproteins presumably work in concert to deliver hemin to the HmuTUV ABC transporter complex for import into the cytoplasm (24, 25, 26), where it is then degraded by the HmuO heme oxidase to release free iron (27). The uptake system also contains HbpA, a Hb receptor that resides on the cell surface which has recently been shown to engage Hb’s α-globin chain (28, 29). Genes encoding the ChtB and ChtC proteins in the system are only present in epidemic strains as a result of a transposon insertion, and based on their related primary sequences are presumably paralogs of HtaB and ChtA, respectively (20, 22).

**Figure 1:**
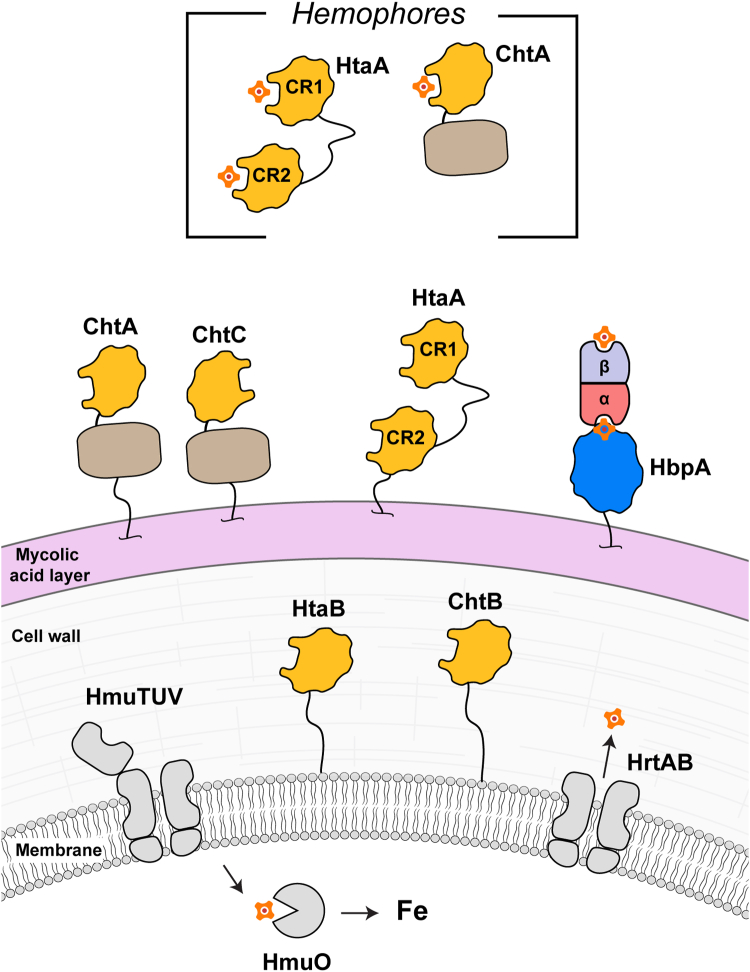
**Schematic of the hemin-acquisition system in *Corynebacterium diphtheriae* (strain 1737).** HbpA binds to hemoglobin or the hemoglobin:haptoglobin (Hb:Hp) complex (28). Hemin released from metHb and other sources is scavenged by a series of hemoproteins that contain conserved region (CR) domains (ChtA, ChtC, HtaA, HtaB, and ChtB) (18, 19, 20, 22). Each of the proteins is presumably embedded in the plasma membrane *via* a C-terminal helix. ChtA and HtaA are also secreted and presumably function as hemophores in the extracellular milieu. Hemin is imported by the HmuTUV complex and degraded by HmuO (27). The HrtAB complex exports excess hemin, which is toxic at high concentrations. The figure shows the three layers of the actinobacterial cell wall, which includes mycolic acid, arabinogalactan, and peptidoglycan layers. The positioning of the proteins is based on cell fractionation and surface shaving experiments (23).

CR domains are present in >2000 species of Actinobacteria (also known as Actinomycetota), a diverse phylum of high GC-content gram-positive bacteria that contains several human pathogens. Crystal structures of CR domains from the soil bacterium *Corynebacterium glutamicum* provided the first insight into their mechanism of hemin binding, revealing that they adopt a β-sandwich fold that coordinates hemin *via* an axial iron-tyrosyl linkage (30, 31). CR domains are structurally distinct from other types of bacterial hemoproteins, including NEAr transporter (NEAT) domains found in Bacillota gram-positive bacteria (32), as well as HasA-like (33, 34) and HmuY-like (35, 36) proteins located in gram-negative bacteria. However, at present little is known about the molecular basis of hemin scavenging by *C. diphtheriae*, which contains a distinct complement of CR containing proteins from *C. glutamicum* (which does not infect humans). Currently only the structure of the CR2 domain from the *C. diphtheriae* HtaA (HtaA^CR2^) protein has been determined (37), and it remains unknown how structural features within CR domains control their affinity for hemin. Here, we report the structural basis through which *C. diphtheriae*’s ChtA and HtaA hemophores scavenge hemin from Hb. Based on mass spectrometry and NMR experiments, we show that their CR domains do not bind to Hb, but that they are still capable of passively acquiring its hemin. Crystal structures of their CR domains bound to hemin reveal distinct pentacoordinate iron binding modes that are confirmed by resonance Raman and electron paramagnetic resonance (EPR) spectroscopy. Quantitative isothermal titration calorimetry (ITC) measurements of hemin binding and targeted mutagenesis shed light on conserved protein-hemin contacts within the CR domain family that modulate their affinity for hemin, and mass spectrometry-based equilibrium partitioning measurements reveal that the CR domains within *C. diphtheriae* exhibit dissociation constants for hemin that range from 850 pM to 22 nM. Overall, our results suggest that *C. diphtheriae* secretes hemophores that passively scavenge hemin from metHb, and that subtle structural differences in their binding pockets modulate hemin affinity, potentially influencing directional heme transfer into the cell.

## Results

### CR domains within ChtA and HtaA passively capture hemin released from Hb

**Figure 2:**
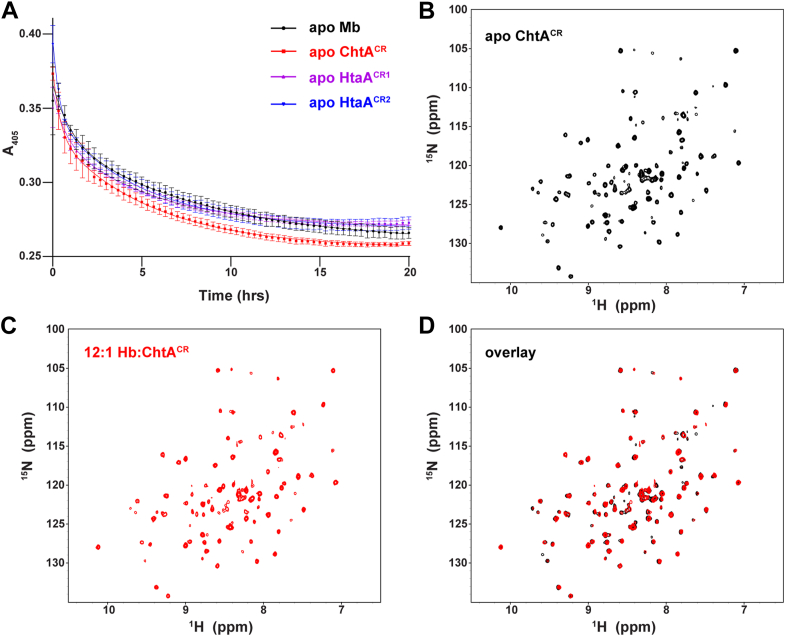
**The hemophore CR domains passively capture hemin from Hb.***A*, UV-visible traces showing hemin transfer from metHb to the hemophore CR domains (ChtA^CR^, HtaA^CR1^, and HtaA^CR2^) or to Mb^H64Y/V68F^. Spectral time courses show the change in Soret band absorbance (405 nm) after mixing metHb (5 μM) with 10-fold molar excess of the apo-acceptor*. Lines* represent the fit to a two-phase exponential decay, and error bars indicate SD (n = 3). *B*–*D*, panels show the ^1^H-^15^N HSQC spectra of ^15^N-labeled apo-ChtA^CR^ either alone (*B*), in the presence of 12-fold molar excess of unlabeled Hb (heme basis) (*C*), and the overlaid spectrum of apo-ChtA^CR^ and its spectrum in the presence of 12-fold molar excess Hb (*D*). No significant spectral changes are observed after the addition of Hb indicating that the proteins do not interact with one another with appreciable affinity. The carbon monoxide bound form of Hb was used, which is more stable and does not release heme to a significant extent during the time course of the experiment. CR, conserved region; HSQC, heteronuclear single-quantum coherence; metHb, methemoglobin.

We wondered if the ChtA and HtaA hemophores are capable of scavenging hemin from Hb. To investigate this question, their isolated CR domains were incubated with metHb and the degree of hemin transfer was determined by monitoring spectral absorbance changes at 405 nm (Fig. 2*A*). When metHb is mixed with a 10-fold molar excess of the apo-form of each CR domain, hemin transfer from metHb is observed; transfer to the CR domains from ChtA (ChtA^CR^, residues K112-G291 of ChtA) and HtaA is observed (HtaA^CR1^, residues S37-G214; HtaA^CR2^, residues G344-G506). The time-dependent ultraviolet-visible spectral changes are biphasic and described by *k*_*fast*_ and *k*_*slow*_ rate constants that describe the rates of hemin dissociation from the alpha and beta subunits of Hb, respectively. These values are compatible with known rate constants of spontaneous hemin release from metHb when apo myoglobin^H64Y/V68F^ is used as a passive hemin scavenger (Table S1) (8, 29, 38, 39). These results are corroborated by native mass spectrometry data that shows hemin moves from metHb to a molar excess of apo-CR domains over the course of hours (Fig. S1).

Prior studies have shown that the CR domains from ChtA and HtaA interact with Hb when it is immobilized and coated on a polystyrene surface (19, 20). However, more recent studies have shown that the use of immobilized Hb can lead to false-positive binding results when hemoproteins are tested. This is presumably because any spontaneously released hemin from Hb is also immobilized and thus serves as ligand for hemoprotein binding. NMR is a useful tool to assess the Hb binding properties of proteins in solution, as complexation with a larger protein such as Hb leads to distinct chemical shift perturbations and significant peak broadening (29, 40). The ^1^H-^15^N heteronuclear single-quantum coherence spectrum of [U-^15^N] ChtA^CR^ is not significantly perturbed when 12-fold molar excess unlabeled Hb is added, indicating that the proteins do not interact with one another with high affinity (Fig. 2, *B*–*D* and S2*D*). Similar results are obtained when Hb is added to either [U-^15^N] HtaA^CR1^ or [U-^15^N] HtaA^CR2^ (Fig. S2, *A* and *B*) indicating that they also fail to bind to Hb with appreciable affinity (29). The limited Hb-dependent spectral perturbations observed for the CR domains is in marked contrast to the HbpA Hb receptor, whose NMR spectrum is substantially broadened when Hb is added (Fig. S2*C*) (29). Thus, the NMR and hemin transfer data collectively show that the isolated CR domains from the ChtA and HtaA hemophores do not directly bind to Hb, but they nevertheless capture its spontaneously released hemin.

### Structures of the ChtA- and HtaA-hemin complexes reveal unique binding pockets

**Figure 3:**
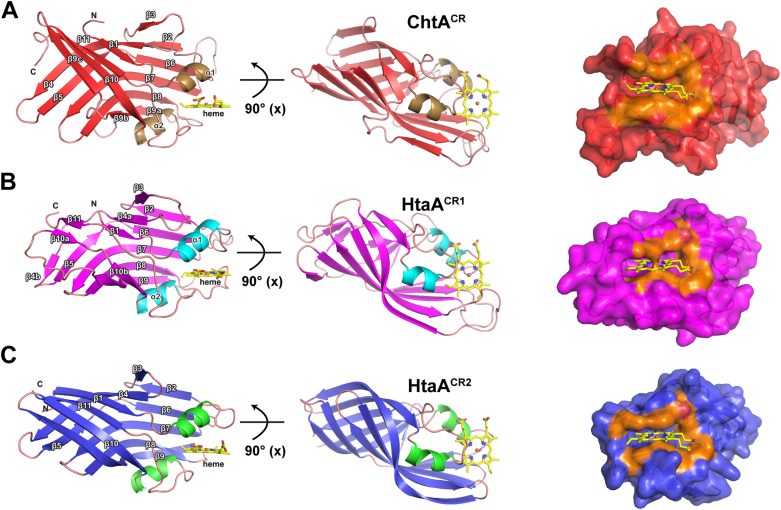
**Crystal structures of the CR-domains from ChtA and HtaA bound to hemin.***A*, *Left*: *ribbon* drawing of the structure of the ChtA^CR^-hemin complex. The α-helices and strands of the β sheet are colored *tan* and *red*, respectively. The heavy atoms of the hemin are shown, with carbon, oxygen, and nitrogen atoms colored *yellow*, *red*, and *blue*, respectively. The iron atom is colored *light orange*. Secondary structural elements are labeled. *Middle*: 90° rotation of the domain. *Right*: surface representation of the ChtA^CR^ protein and heme pocket, with hemin interacting residues colored *dark orange*. (*B*) As in (*A*) for the HtaA^CR1^-hemin complex. The color scheme is identical to (*A*) except that the α-helices and strands of the β sheet are colored *cyan* and *magenta,* respectively. (*C*) As in (*A*) for the previously solved HtaA^CR2^-hemin complex (37) (PDB: 8SMU). The color scheme is identical to (*A*) except that the α-helices and strands of the β sheet are colored *green* and *blue*, respectively. CR, conserved region; PDB, Protein Data Bank.

**Figure 4:**
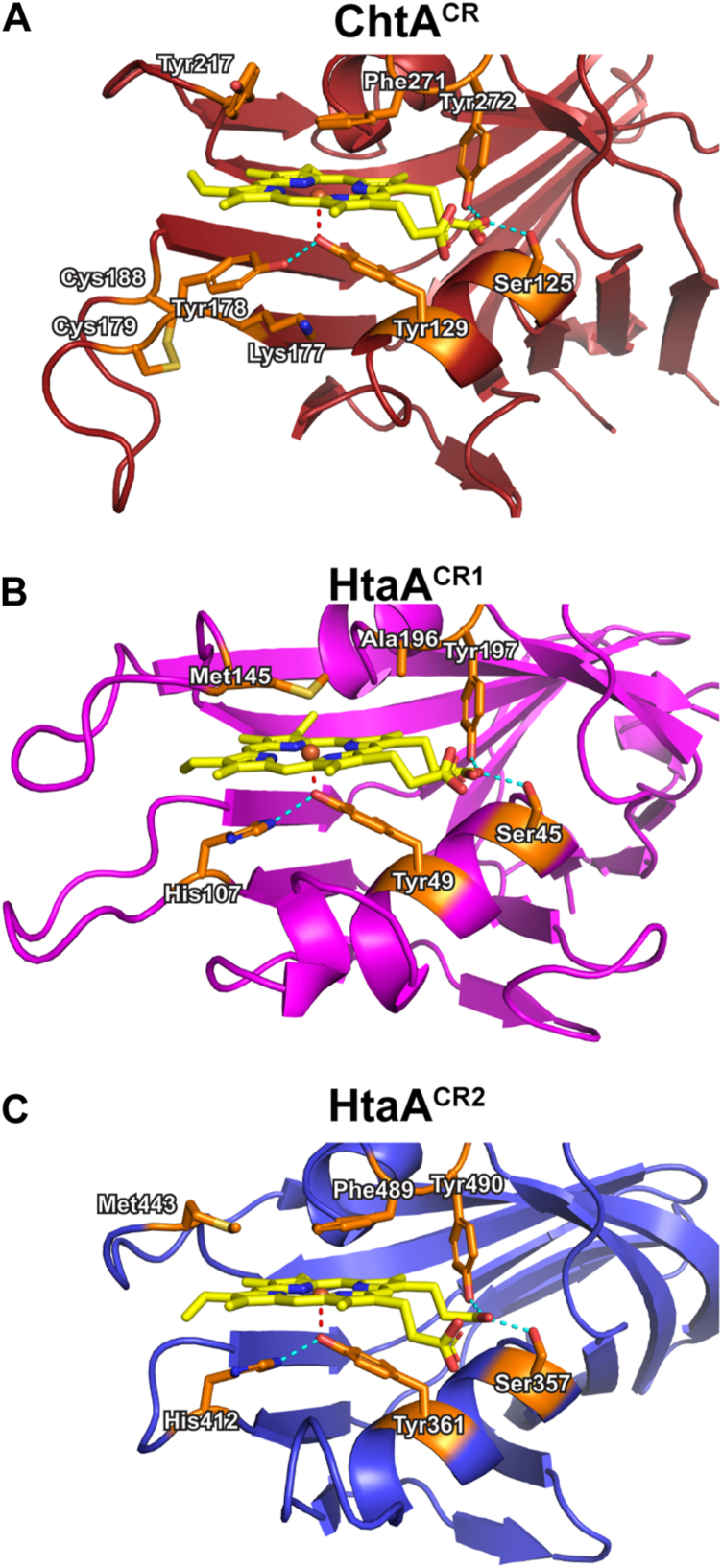
**Hemin interactions with ChtA and HtaA.***A*, expanded view of the hemin binding pocket in the ChtA^CR^-hemin complex. The protein is rendered in *ribbon* format and colored *red*. The heavy atoms of the hemin are shown with carbon, oxygen, and nitrogen atoms colored *yellow*, *red*, and *blue*, respectively. The side chains of hemin-interacting residues are shown as *orange sticks* and labeled. Potential hydrogen bond interactions are colored in *light blue* and the iron-tyrosyl linkage is in *red*. *B*, expanded view of the HtaA^CR1^-hemin complex colored as in panel (*A*) except the protein backbone is colored *magenta*. *C*, expanded view of the previously solved HtaA^CR2^-hemin complex (37) colored as in panel (*A*) except the protein backbone is colored *blue*. CR, conserved region.

**Figure 5:**
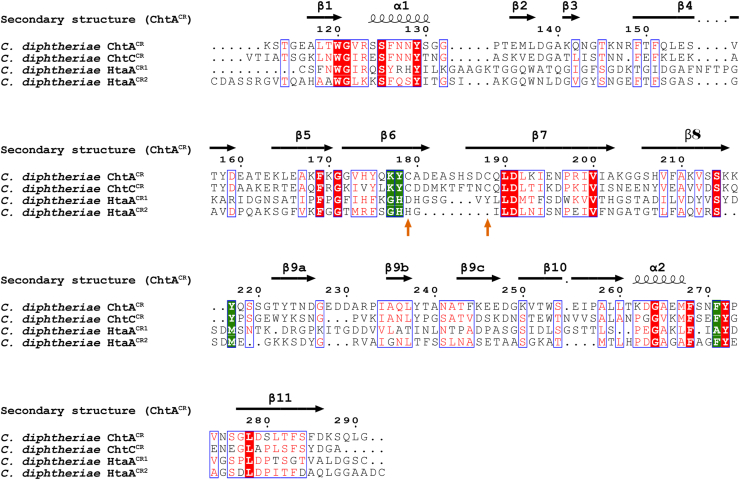
**Primary sequence alignment of the hemophore and surface displayed CR domains in *Corynebacterium diphtheriae*.** Residue numbering and secondary structure annotation is based on ChtA. The alignment highlights universally conserved residues (*dark red boxes*), including the axial tyrosine (Tyr129) and propionate-interacting residues (Ser125 and Tyr272). Partially conserved residues are enclosed in a *box* and presented in *red* text. Residues involved in hemin binding that differ between the CR domains are shaded *green*. Cysteine residues that form a disulfide-bond in ChtA are indicated by *orange arrows*. CR, conserved region.

To better understand how the *C. diphtheriae* hemophores engage hemin, we determined high-resolution crystal structures of ChtA^CR^ and HtaA^CR1^ in complex with hemin. The ChtA^CR^-hemin complex crystallized in the C222_1_ space group, and its structure was determined at 1.63 Å resolution using molecular replacement methods (Fig. 3*A*, Table S2). Two copies of the complex are present in the asymmetric unit (ASU) and are well-defined by continuous electron density. ChtA^CR^ is composed of 11 β-strands that form a sandwich constructed from a long antiparallel β-sheet composed of the β2 and β5- β9a strands, and a shorter antiparallel β-sheet composed of strands β1, β4, β10, and β11. Hemin is nestled within a groove located near the end of the β-sandwich, partially ensconced between helices α1 and α2. The side chain of Tyr129 within the α1 helix axially coordinates hemin’s iron atom and forms π-π stacking interactions with its A and D-pyrrole rings (Fig. 4*A*). This face of the hemin is also contacted by a “helper” tyrosine residue (Tyr178 within the β6-β7 loop), which is positioned to form hydrogen bonding interactions with the metal-coordinating Tyr129 residue. Presumably, hydrogen bonding between these residues facilitates deprotonation of the apo-form of the protein by decreasing the pKa of the axial Tyr129, which may favor binding to ferric iron within hemin. The side chain ε-amino group in K177 is also positioned within ∼3.0 Å of Y178 hydroxyl and thus may further stabilize its positioning *via* hydrogen bonding. This Tyr-Tyr interaction has not been observed previously in the structures of other CR domains, which instead employ “helper” histidine residues to stabilize the metal-coordinating tyrosine (30, 37). The structure of the β6–β7 loop housing the helper Tyr178 in the ChtA^CR^ is also distinct, as it is considerably longer than the corresponding loops in other CR domain structures, and it contains a unique disulfide bond between Cys179 and Cys188. Residues in the β6-β7 loop exhibit slightly higher than average crystallographic B-factors suggesting that the loop exhibits elevated mobility. Residues within the α2-β11 and β8-β9 loops contact the hemin face positioned opposite the tyrosyl-metal bond. In particular, the phenyl ring of Phe271 (α2-β11 loop) is stacked over hemin’s C and D pyrrole groups limiting their solvent exposure. The side chain of Tyr217 (β8-β9 loop) is also positioned nearby, but the phenyl ring of Phe271 obstructs its interactions with hemin. The Phe271 capping residue forms a T-like interaction with Tyr217 that may stabilize its interactions with hemin, as the side chains of these aromatic residues are arranged with a plane angle of ∼60° and a centroid-centroid distance of ∼5.2 Å (41, 42). The bound hemin molecule projects one of its propionate groups into the solvent, while the other propionate is contacted by the hydroxyl groups of Ser125 (helix α1) and Tyr272 (α2-β11 loop), which are highly conserved in CR domains (Figs. 5, S3 and S4) A representative unbiased omit map for the bound hemin ligand is shown in Fig. S5.

We next investigated how the HtaA protein captures hemin. In previous work, we determined the structure of the C-terminal HtaA^CR2^-hemin complex (37), but how the N-terminal CR1 domain binds hemin is not well understood. The crystal structure of the HtaA^CR1^-hemin complex was determined at 1.88 Å resolution (Fig. 3*B*, Table S2). It crystallized in the C2 space group with ten complexes in the asymmetric unit. Atypical intensity statistics indicative of crystal twinning were observed during data processing and structure refinement stalled at a high R_work_/R_free_ ratio. This issue was resolved by the application of a twin operator (h, -k, -l; twin fraction α = 0.5), whose use is supported by a comparison of the R_merge_ values between twin-related reflections for both *I*_*calc*_ and *I*_*obs*_ (Fig. S6) (43). In the final structure, complexes containing protein chains A to E are well defined by continuous electron density, while complexes containing chains F to J exhibit weaker and more discontinuous density. Despite this, minimal structural differences are observed between all chains in the asymmetric unit, and their heavy atom coordinates can be superimposed with a rmsd of 0.26 to 0.58 Å. In each complex, the HtaA^CR1^ domain adopts a canonical CR fold that somewhat resembles ChtA^CR^, but notable differences occur in the positioning of the strands at the terminal end of the beta sandwich, with strands β4 and β10 being discontinuous in HtaA^CR1^ (in HtaA^CR1^ the strands are referred to as β4a and β4b, and β10a and β10b, respectively). Overall, protein residues spanning strands β1 to β11 in the two CR domains can be overlaid with an rmsd of 3.45 to 3.62 Å. As in the structure of the ChtA^CR^-hemin complex, a tyrosine located in the α1 helix (Tyr49) is an axial ligand to hemin’s iron, and conserved serine (Ser45 in helix α1) and tyrosine (Tyr197 in the α2-β11 loop) residues are positioned to jointly hydrogen bond to one of hemin’s propionate groups (Fig. 4*B*). However, beyond these conserved features, the ChtA^CR^ and HtaA^CR1^ hemophores exhibit large differences in their mode of hemin binding. For example, in HtaA^CR1^ a “helper” histidine side chain located in the β6-β7 loop (His107) is positioned to hydrogen bond to the axial tyrosine ligand that coordinates the metal, whereas in ChtA^CR^ a second tyrosine residue (Tyr178 in ChtA^CR^) fulfills this role. In addition, as compared to ChtA^CR^, the β6-β7 loop in HtaA^CR1^ loop that houses the helper residue lacks a disulfide bond. Protein-hemin contacts on the opposite face of the protoporphyrin ring also differ as the conformation of the β8-β9 loop in HtaA^CR1^ is significantly longer than in ChtA^CR^, and it harbors a methionine residue (Met145) that is positioned near hemin’s iron (the Sδ-Fe distance is ∼2.9 Å). Its close approach to the iron is made possible by differences in the α2-β11 loop, since unlike ChtA^CR^ which contains a phenylalanine in the α2-β11 loop that caps hemin access from the solvent (Phe271 in ChtA^CR^), in HtaA^CR1^ the phenylalanine residue is replaced with a smaller, less intrusive alanine side chain (Ala196). A representative unbiased omit map for the bound hemin ligand is shown in Fig. S5.

The structure of the previously characterized C-terminal HtaA^CR2^ from *C. diphtheriae* is most similar to the N-terminal HtaA^CR1^ domain presented here, and can be overlaid with a backbone rmsd of 1.95 to 2.2 Å. HtaA^CR2^ positions His412 in the β6-β7 loop at an equivalent position as His107 in HtaA^CR1^’s, though the loop itself is noticeably shorter (Fig. 3*C*). Interestingly, both CR domains within the HtaA hemophore contain methionine residues at similar sites within their β8-β9 loops; Met145 and Met443 in HtaA^CR1^ and HtaA^CR2^, respectively. However, as compared to HtaA^CR1^, the Met443 residue in HtaA^CR2^ is positioned more distal to the metal center because the iron is capped by Phe489 (the Sδ-Fe distance in the HtaA^CR2^-hemin complex is ∼5.4 Å) (Fig. 4*C* and 5).

### Resonance Raman and EPR spectroscopy experiments probe the coordination environment of the ChtA and HtaA hemophores

**Figure 6:**
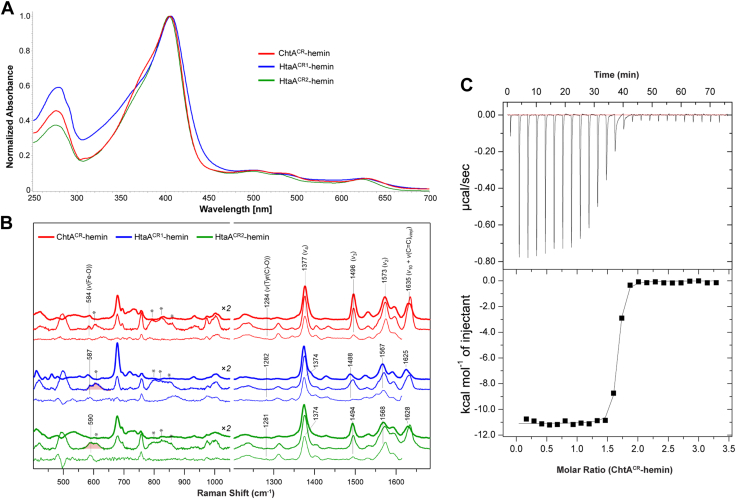
**Spectroscopic and calorimetric measurements of hemin binding to CR domains.***A*, representative UV-visible spectroscopic traces for the 250 to 700 nm region for ChtA^CR^-hemin (*red*), HtaA^CR1^-hemin (*blue*), and HtaA^CR2^-hemin (*green*) protein complexes. The clear Soret peak at 405 nm is indicative of coordinated hemin. *B*, resonance Raman spectra (77 K) of ChtA^CR^-hemin, HtaA^CR1^-hemin, and HtaA^CR2^-hemin in the 400 to 1700 cm^−1^ region. All spectra were collected with 407 nm (*thick lines*), 457 nm (*medium thickness*), or 488 nm (*thin lines*) excitation. *C*, representative isothermal titration calorimetry (ITC) data showing hemin binding to ChtA^CR^. The binding isotherm was obtained by injecting apo-protein (495 μM) into a cell containing hemin chloride (30 μM) at a constant temperature of 25 °C. (*top*) time-course of the titration (*black*) and baseline (*red*). (*bottom*) Fitted integration of the isotherm. Thermodynamic parameters were obtained by nonlinear regression to a one-site binding model with the program ORIGIN. CR, conserved region.

To gain insight into how the hemophores engage hemin in solution, we recorded the UV-visible absorbance and resonance Raman (rR) spectra of the isolated ferric HtaA^CR1^, HtaA^CR2^, and ChtA^CR^ domains. Consistent with each protein coordinating hemin *via* an axial tyrosine linkage, their UV-visible spectra contain a Soret maximum at 405 nm, as well as bands at 502, 537, and 624 nm. The bands for ChtA are shifted slightly to 406, 497, 538, and 625 nm (Fig. 6*A*).

RR spectra of ChtA^CR^, HtaA^CR1^, and HtaA^CR2^ were obtained with multiple excitation wavelengths to enhance distinct porphyrin and iron-tyrosinate vibrational modes, which were used to evaluate the solution-phase oxidation state, spin state, and coordination number, along with variations across the three systems (Fig. 6*B*, Table S3). In the high-frequency region reflecting the porphyrin in-plane skeletal modes (1100–1650 cm^−1^), the most intense band arises near 1375 cm^−1^ for ChtA^CR^-hemin, HtaA^CR1^-hemin, and HtaA^CR2^-hemin with Soret excitation. This band represents the symmetric core breathing mode, *v*_4_, which is a known oxidation state marker that occurs near 1370 cm^−1^ for ferric ions (44). Hence, this confirms the Fe(III) state persists in solution. The *v*_3_ mode observed near 1490 cm^−1^ has been used as a spin-state and coordination number indicator (31, 45, 46, 47). For hemins with an axial tyrosine ligand, the *v*_3_ band of five-coordinate (5c), high-spin (HS) hemins falls within the range of 1487 to 1494 cm^−1^, higher than those of six-coordinate (6c) HS hemins (1478–1482 cm^−1^) (45, 46, 48). The *v*_10_ band frequency is considered another spin-state marker, occurring within a frequency range of 1622 to 1626 cm^−1^ for 5c HS hemins and 1605 to 1612 cm^−1^ for 6c HS hemins (46, 48, 49, 50). In this work, the *v*_10_ modes from the hemoproteins appear at slightly higher frequencies than previously observed (1632–1636 cm^−1^). Finally, the intensity ratio of I(*v*_4_)/I(*v*_3_), another indicator of the spin-state and coordination number of a hemin, was examined. For ChtA^CR^-hemin, HtaA^CR1^-hemin, and HtaA^CR2^-hemin, intensity ratios of 1.3, 6.2, and 2.2, respectively, are obtained with 407 nm excitation. For 5c HS hemoproteins, relatively low I(*v*_4_)/I(*v*_3_) ratio (∼1.5) are typically reported (46). However, it should be noted that the intensity of the *v*_3_ band varies dramatically among the hemoproteins and across excitation wavelengths, and slight changes in the relative energies between the absorption maxima and excitation wavelength can have dramatic effects on intensity, diminishing the robustness of this marker. Considering the collective vibrational markers described herein, the data suggest that the hemins from ChtA^CR^, HtaA^CR1^, and HtaA^CR2^ are pentacoordinate, HS ferric centers. Thus, even though the structural data reveal that HtaA^CR1^ presents a methionine side chain near the metal center (Fig. 4*B*), in solution no significant Sδ-Fe coordinating interactions occur.

Identification of vibrational modes reflecting the heme-tyrosinate coordination was also pursued. Bands between 584 and 590 cm^−1^ were observed in the samples, with higher intensities at longer excitation wavelengths. These bands are assigned to the *v*(Fe–O) stretching mode coupled with a phenolate ring vibration, which is known to occur in the 580 to 600 cm^−1^ range (46, 47, 48, 49). Another Tyr-derived band has been observed in prior reports around 1270 to 1310 cm^−1^, which is mainly attributed to the C–O stretching mode (51). The corresponding band appears, albeit weakly, at 1284, 1282, and 1281 cm^−1^ from ChtA^CR^-hemin, HtaA^CR1^-hemin, and HtaA^CR2^-hemin, respectively. The similarity in C-O frequency across the three proteins suggests a similar coordination motif to the Fe center, indicative of comparable H-bonding strength to the axial tyrosinate from both the “helper” tyrosine or “helper” histidine residues.

To confirm the spin states of the ChtA^CR^-, HtaA^CR1^-, and HtaA^CR2^-hemin complexes, continuous wave EPR spectra were collected (Fig. S7). Characteristic features of HS, 5c heme proteins include features at g∼6.1 and g∼2, with additional rhombicity introduced upon tyrosinate coordination that results in splitting of the low-field feature to g∼6.6 and 5.3 and additional satellite features at g∼6 (52, 53, 54, 55). The spectra of ferric ChtA^CR^-hemin, HtaA^CR1^-hemin, and HtaA^CR2^-hemin are similar to previously reported spectra of other tyrosinate-bound heme proteins, such as the H93Y mutant of myoglobin (52). Importantly, there are no signals that would correspond to low-spin ferric heme (53, 54), which would be expected in a hexacoordinate Tyr-Met coordination motif. The absence of such signals supports the conclusion from resonance Raman that, despite the proximity, the methionine residue remains unbound to the iron center in HtaA^CR1^.

### ITC measurements of hemin binding to ChtA^CR^

To gain insight into the thermodynamics underlying CR-hemin interactions, ITC was used to investigate hemin binding to ChtA^CR^ (Fig. 6*C*). This analysis revealed that ChtA^CR^ binds hemin with a dissociation constant (K_D_) equal to 22 ± 5 nM (ΔG° = −10.5 kcal mol^−1^) in a process that is driven by favorable enthalpic changes (ΔH° = −11 kcal mol^−1^) and opposed by a much smaller change in entropy (TΔS° = 0.5 kcal mol^−1^) (Table 1). Characteristic of a high affinity interaction, the slope of the enthalpic transition is quite steep, with fewer points to define the line through the transition point from which K_A_ (and therefore K_D_) are fit. Despite this, the triplicate measurements show relatively tight standard error, and the magnitude of the measured affinities is consistent with previously reported values for other bacterial hemin binding proteins (see Discussion section). As expected, a ChtA^CR^ variant containing a Y129A substitution that eliminates the axial tyrosine that coordinates iron exhibits >25-fold decrease in binding affinity. In contrast, eliminating the helper tyrosine residue by introducing a Y178A substitution has a more modest effect, reducing affinity only ∼2-fold. Interestingly, introduction of a Y178H alteration to create a H178 (helper)-Y129 (axial) unit that resembles what is present in all other previously studied CR domains did not restore ChtA^CR^’s affinity for hemin. Notably, in the structure of the ChtA^CR^-hemin complex a third residue interacts with the Y178 (helper)-Y129 (axial) unit; the amine group from Lys177 (strand β4) is positioned to hydrogen bond with the Y178 (helper) side chain (Fig. 4*A*). In the structures of the HtaA CR domains this lysine is replaced with a glycine residue (G106 and G411 in HtaA^CR1^ (Fig. 4*B*) and HtaA^CR2^ (Fig. 4*C*), respectively), and no hydrophilic side chains are positioned near their histidine (helper)-tyrosine (axial) units (Fig. 5). To investigate the role of Lys177 in binding, we determined the hemin affinities of both single K177G and double K177G + Y178H variants. Interestingly, the K177G protein still exhibits reduced affinity, while K177G + Y178H substitutions function synergistically to restore hemin affinity to near WT levels (K_D_ = 27.1 ± 9.0 nM). Representative thermograms and fits for all mutants in Table 1 are shown in Fig. S8. Collectively, the data suggest that context-dependent stereochemical interactions involving the helper tyrosine in ChtA^CR^ are needed for it to stabilize hemin binding by the axial tyrosine.

**Table 1:** Isothermal titration calorimetry measurements of hemin binding to ChtA^CR^

Protein	K_D_ (nM)^a^	ΔG° (kcal/mol)	ΔH° (kcal/mol)	−TΔS° (kcal/mol)^b^	N^c^
WT	22.4 ± 5.3	−10.5	−10.5 ± 0.3	0.5	1.8
Y178H	54.8 ± 1.9	−9.7	−12.1 ± 0.4	2.4	1.6
K177G	23.6 ± 0.8	−10.5	−14.5 ± 0.3	4	1.4
K177G + Y178H	27.1 ± 9.0	−10.3	−9.9 ± 0.2	−0.5	1.8
Y178A	46.6 ± 9.6	−10.1	−6.4 ± 0.3	−3.7	1.7
Y129A	>500	<−8.5	ND^d^	ND	1.1

a Values reported for average of three replicates.

b Temperature of 25 °C (298 K).

c n refers to molar ratio of protein/hemin.

d ND, magnitude of enthalpy change not available due to poor pre-enthalpic transition data.

### CR domains in *C. diphtheriae* exhibit a range of affinities for hemin

**Figure 7:**
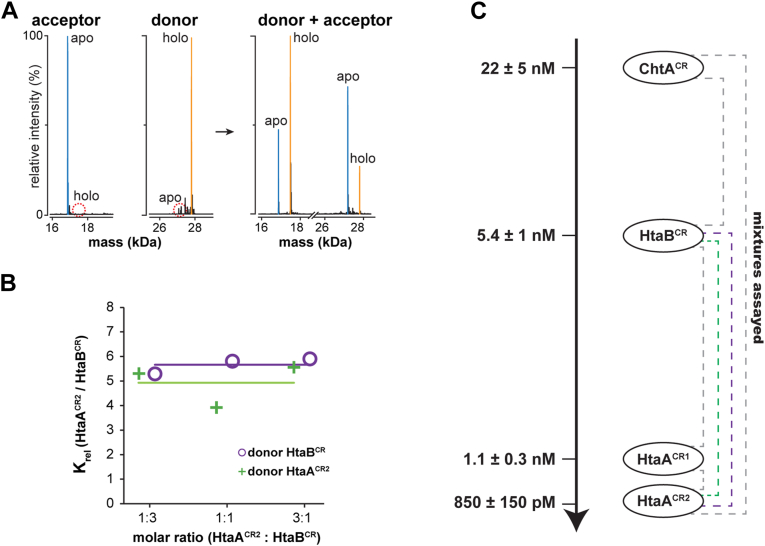
**Hemin partitioning between *Corynebacterium diphtheriae* CR domains.***A*, representative native ESI-MS spectra showing hemin partitioning between HtaB^CR^ (donor) and HtaA^CR2^ (acceptor). The proteins were mixed and the transfer process equilibrated. The MS spectrum on the *right* reveals the degree of hemin partitioning, as determined by measuring the intensities of the apo- and holo-forms of each protein. The reaction reaches equilibrium within 4 h, with little difference in the spectra of the same sample after 24 h. *B* plot showing values of the relative hemin binding constant (K_rel_) for the HtaB^CR^ and HtaA^CR2^ proteins. Values of K_rel_ were obtained from six independent reactions in which the hemin acceptor:donor ratios were varied. Reactions in which HtaB^CR^ was the hemin donor and acceptor are indicated by *purple circles* and *green plus* signs, respectively. The data show that consistent measurements of K_rel_ are obtained for all experiments (the line indicates the average value of K_rel_ that was obtained from the different experiments). *C*, graph showing the relative affinities and estimated hemin dissociation constants for the ChtA^CR^, HtaB^CR^, HtaA^CR1^, and HtaA^CR2^ proteins obtained from a total of 21 independent hemin partitioning reactions (Table S4). For ChtA^CR^, K_D_ for hemin was determined by ITC, while for the other proteins K_D_ values were obtained using the nano ESI-MS K_rel_ measurements. Dotted lines represent the partitioning experiments that were performed (data for experiments involving HtaA^CR2^ and HtaB^CR^ are colored as in panel (*B*)). CR, conserved region; ESI-MS, electrospray ionization mass spectrometry; ITC, isothermal titration calorimetry; MS, mass spectrometry.

The *C. diphtheriae* hemin-uptake system contains six CR domains that are located at distinct sites outside the cell (Fig. 1). Except for ChtA^CR^*,* attempts to measure the hemin affinities of CR domains by ITC proved unsuccessful because their apo-forms either aggregated or because they exhibited binding isotherms with sharp enthalpic transitions that prevented numerical definition of their K_D_ values. We therefore determined the relative hemin affinities of several *C. diphtheriae* CR domains using native electrospray ionization mass spectrometry (nESI-MS), which can be performed at dilute concentrations to reveal the relative amounts of the apo- and holo-forms of a protein (37). The degree of hemin partitioning between 4 *C. diphtheriae* CR domains was studied: (1) ChtA^CR^, (2) HtaA^CR1^, (3) HtaA^CR2^, and (4) HtaB^CR^ (HtaB, residues of 30–273). Together, they represent nearly the full complement of *C. diphtheriae’s* CR domains (only the domains from ChtB and ChtC are missing, but they are expected to have similar binding properties as their paralogs, HtaB and ChtA, respectively). In the partitioning experiment, pairs of CR domains in either their apo (acceptor) or hemin bound forms (donor) are mixed with one another at different stoichiometric ratios (donor:acceptor ratios ranging from 1:3, 1:1–3:1). The proteins are then incubated for 4 h to enable the hemin transfer reaction to come to equilibrium and the degree of partitioning measured by native ESI-MS (Fig. 7). As described in the Experimental Procedures section, the ratio of the equilibrium concentrations of the holo- and apo-forms of each protein is related to a relative hemin binding constant (K_rel_), a thermodynamic constant that is itself equal to the ratio of each protein’s K_D_ for hemin (56). Importantly, the measured value of K_rel_ should be independent of the initial concentrations of the hemin donor and acceptor, enabling it to be redundantly determined when different stoichiometric ratios are used. Representative partitioning data for a reaction containing HtaB^CR^ (donor) and apo-HtaA^CR2^ (acceptor) clearly shows that HtaA^CR2^ has higher affinity for hemin (Fig. 7, *A* and *B*). Similar measurements were made for the following protein pairs: HtaA^CR2^/HtaB^CR^, HtaA^CR1^/HtaB^CR^, HtaA^CR2^/HtaA^CR1^, ChtA^CR^/HtaA^CR2^, and ChtA^CR^/HtaB^CR^ (Table S4). This analysis yielded the following order of hemin affinities, from weakest to strongest: ChtA^CR^ < HtaB^CR^ < HtaA^CR1^ ∼ HtaA^CR2^ (Fig. 7*C*). Overall, they exhibit a ∼25-fold variation in binding strength and using the ITC determined value for ChtA^CR^ as a reference, their estimated K_D_ values for hemin are: ChtA^CR^ K_D_ = 22 ± 5 nM, HtaB^CR^ K_D_ = 5.4 ± 1 nM, HtaA^CR1^ K_D_ = 1.1 ± 0.3 nM, and HtaA^CR2^ K_D_ = 850 ± 150 pM.

## Discussion

Heme is the most abundant source of iron in the human body and is actively acquired by bacteria during infections. Epidemic isolates of *C. diphtheriae* capture heme-iron using CR domains located within five proteins (ChtA, ChtC, HtaA, HtaB, and ChtB) (18, 19, 20, 22) (Fig. 1). Recent studies indicate that the ChtA and HtaA proteins are secreted into the extracellular environment (19, 22, 23), and it has been demonstrated that cells in which either *chtA* or *htaA* are genetically deleted are impaired in their ability to use Hb or the Hb:Hp complex as an iron source (19, 22). In this study, we sought to gain insight into how these hemophores acquire hemin from metHb. Our results indicate that the secreted CR domains do not interact with Hb, but nevertheless capture hemin from this protein that is first passively released into the solvent (Fig. 2). The finding that they do not interact with metHb when tested by solution state NMR (Fig. 2, *B*–*D*) is in conflict with previous reports that detected interactions with immobilized metHb (19, 20). The origin of this discrepancy is not known, but binding studies using immobilized metHb can cause false-positive results because hemin released from metHb is also immobilized and thus a potential ligand for hemoproteins (40). Our NMR studies would seem to rule out high affinity protein-protein binding, since no significant spectral perturbations are observed in the CR domains even when Hb is present at 12-fold molar excess. However, it remains conceivable that other amino acid segments from the full-length proteins may mediate Hb binding.

To learn how the hemophores engage hemin, we determined structures of the ChtA^CR^-hemin and HtaA^CR1^-hemin complexes by X-ray crystallography, which in combination with a previously reported structure of HtaA^CR2^-hemin complex (37), provides a comprehensive view of hemin binding. The CR domains in ChtA and HtaA adopt related β-sandwich folds and coordinate hemin’s iron *via* conserved tyrosine residues that are located in helix α1 (Y129, Y49, or Y361, in ChtA^CR^, HtaA^CR1^, or HtaA^CR2^, respectively). The structures of HtaA^CR1^ and HtaA^CR2^ are closely related as their protein backbone atoms can be superimposed with a rmsd of 1.95 to 2.2 Å. In contrast, the stacked β-sheets positioned distal to the hemin pocket in ChtA^CR^ are splayed leading to larger structural differences in comparison to the HtaA^CR1^ and HtaA^CR2^ domains to (rmsd values of 3.45–3.62 and 3.02–3.19 Å, respectively) (Fig. 3). In addition to the metal coordinating tyrosine residue, the domains also make similar contacts with hemin’s buried propionate arm *via* conserved serine and tyrosine residues located in α1 helix and the α2-β11 loop, respectively (S125 and Y272, S45 and Y197, or S357 and Y490, in ChtA^CR^, HtaA^CR1^ or HtaA^CR2^, respectively) (Fig. 4). These amino acids are highly conserved and similar hemin interactions have been observed in crystal structures of homologous CR domain-hemin complexes from *C. glutamicum* (30) (Figs. S3 and S4). Beyond these similarities, significant variations occur in their mode of hemin binding. In particular, in ChtA^CR^ the metal coordinating tyrosine (Y129) is stabilized by a unique, second “helper” tyrosine residue (Y178) that is located in the β6-β7 loop; in HtaA and all other previously characterized CR domains the helper residue is a histidine (30, 37). In ChtA contacts to the hemin metal are presumably further indirectly stabilized by K177, as its ε-amino group is positioned 3.0 Å from the Y178 hydroxyl for potential favorable electrostatic interactions. Targeted alteration of these residues in ChtA^CR^ reveals that the axial tyrosine is critical for hemin binding, while exchanging the helper tyrosine residue with alanine reduces affinity ∼2-fold. Interestingly, our mutational analysis reveals that the environment surrounding the helper residue alters its effect on hemin affinity. This is because simply replacing the helper tyrosine in ChtA^CR^ with a histidine residue that is commonly found in other CR domains does not restore hemin affinity, in agreement with previous qualitative reports (20). This is likely due to the presence of K177 in ChtA, which is unique to this CR domain as the equivalent residue preceding the axial ligand is a glycine in the other CR domains that use a histidine to coordinate heme’s iron atom. Indeed, only when both "axial-helper" pair residues are swapped for those found in other CR domains (*i.e.* K177G + Y178H) is WT affinity recovered, suggesting that these tandemly arranged residues in the β6-β7 loop work in concert to stabilize the anionic form of the axial tyrosine.

As compared to HtaA and other previously characterized CR domains, the β6-β7 loop housing K177 and Y178 in ChtA^CR^ is also structurally unique, as it contains a disulfide-bond and is longer than the analogous loops found in other types of CR domains (Fig. 5). The ChtA hemophore and its paralog ChtC appear to only be present in pathogenic Corynebacterium species (*e.g. C. diphtheriae, Corynebacterium ulcerans,* and *Corynebacterium pseudotuberculosis*), with the closely related ChtC likely engaging hemin in a similar manner to ChtA^CR^, as both proteins share a high level of primary sequence homology (Fig. S4).

Although structural and spectroscopic data reveal that the HtaA and ChtA hemophores form pentacoordinate interactions with hemin, varied interactions with the face opposing the tyrosyl-metal bond may modulate the ability of their CR domains to transfer hemin (Figs. 6*B* and S3). In the structures of the ChtA^CR^- and HtaA^CR2^-hemin complexes the opposing face is “capped” by a phenylalanine side chain, which sterically occludes access to the metal center. In marked contrast, in HtaA^CR1^, a methionine side chain (Met145) located in the β8-β9 loop closely approaches the metal in the structure of its hemin complex. However, consistent with the results of rR and EPR experiments the distance of approach is not sufficiently close to enable metal coordination (the Fe-S_Met_ distance is 2.9 Å) (Figs. 4*B* and 6*B*). The functional relevance of methionine metal capping in HtaA^CR1^ remains unknown, but it is notable that S_Met_ interactions with Fe(II) are stabilizing, such that the hemin bound to HtaA^CR1^ may have a higher reduction potential as compared to the Phe-capped hemin molecules within ChtA^CR^ and HtaA^CR2^. This could have consequences for the directionality of heme transfer if the redox potential and the K_D_ of transfer were coupled and remains to be investigated. Based on their primary sequences, the CR1 domains located in pathogenic Corynebacterium species (*e.g. Corynebacterium jeikium, Corynebacterium pseudotuberculosis,* and *Corynebacterium ulcerans*) may form similar methionine hemin capping interactions, as they also contain residues homologous to Met145 in HtaA^CR1^ and replace the bulky Phe cap observed in HtaA^CR2^ and ChtA^CR^ with unobtrusive residues like Ala or Gly (Fig. S3).

ITC measurements provide the first-ever insight into the energetics that underpin hemin-CR domain interactions, revealing that ChtA^CR^ binds hemin with a K_D_ of 22 ± 5 nM through favorable enthalpic changes that presumably accompany axial bond formation (ΔH^0^ of binding equals −10.5 kcal mol^−1^). In contrast, a slightly unfavorable entropic change occurs that may be a consequence of conformational ordering of the binding pocket. Two types of hemophores have been identified in gram-positive bacteria: CR domains present in Actinobacteria, and NEAT domains that are found in *Staphylococcus aureus* and other species within the Bacillota phylum (also known as the Firmicutes phylum) (32). Interestingly, ITC studies indicate that NEAT domains have similar affinities for hemin as compared to ChtA^CR^, and that their binding is also driven by similar standard state enthalpic changes. For example, *S. aureus* IsdH and IsdA NEAT domains bind hemin with K_D_ values of 34 ± 8 and 14 ± 4 nM, respectively, and ΔH^0^ values of −10.2 and −10.8 kcal mol^−1^, respectively (57, 58). This is consistent with both types of bacterial hemoproteins forming pentacoordinate iron-tyrosyl linkages to hemin’s iron atom, and crystal structures of their complexes that reveal that they bury a similar amount of hemin’s surface area (530 Å^2^ for ChtA^CR^ *versus* 534 and 538 Å^2^ for IsdH and IsdA, respectively). Interestingly, both NEAT and CR domains exhibit noncanonical hemin binding stoichiometries when investigated by ITC. The hemin binding stoichiometry parameter for ChtA^CR^ suggests that 1 to 2 copies of ChtA^CR^ bind to a single hemin (n = 1.79 ± 0.166, 5 replicates), whereas previously reported ITC data yielded an inverse binding stoichiometry for NEAT domains, ∼2 hemin molecules bound to 1 NEAT domain (57, 58). We do not believe that this difference is significant, since based on structural and native mass spectrometry data both types of domains bind a single hemin molecule. For the case of the ChtA^CR^ protein, an observed protein:hemin ratio in excess of one could result from protein aggregation or degradation that decreases the amount of functional protein present in the ITC experiment. In particular, the methyl ethyl ketone method used to remove hemin from ChtA^CR^ prior to performing the ITC employs harsh conditions, which would lead to an elevated stoichiometry parameter. Although it cannot be excluded, it seems less likely that protein aggregation causes reduced functionality as only the monomeric form of apo-ChtA^CR^ is observed in analytical gel filtration experiments performed at concentrations that exceed those present in the ITC reaction cell or injection syringe (Fig. S9). Moreover, control ITC experiments in which apo-ChtA^CR^ is injected into a buffer solution yield only small magnitude heat changes incompatible with concentration-dependent dissociation of oligomeric species. The inverted binding stoichiometry previously observed for NEAT domains (two hemins per protein) may result from the ability of the NEAT domain fold to engage dimeric forms of hemin. In this binding mode, two hemins would be bound to the protein, one hemin coordinated *via* an iron-tyrosyl linkage to the NEAT domain, with a second hemin associated with the first *via* π-π stacking or (Fe-O-Fe) μ-oxo bridges. In contrast, based on the structures of CR-hemin complexes, dimeric hemin binding seems less unlikely due to steric effects. Regardless of the differences in their ITC derived hemin binding stoichiometries, X-ray crystallography structural data indicate that CR and NEAT domains adopt fundamentally different protein folds that bind a single hemin molecule *via* a tyrosyl-metal bond, which based on our ITC experiments possess similar hemin affinities and energetic signatures.

In summary, our results reveal that the *C. diphtheriae* ChtA and HtaA hemophores scavenge hemin after it is spontaneously released from Hb. Their CR domains bind hemin *via* a conserved iron-tyrosyl coordination bond (30, 31, 37), but otherwise exhibit substantial differences in how they engage hemin, including how the axial tyrosine is stabilized and hemin’s metal is masked. ITC measurements employing ChtA^CR^ demonstrate its hemin binding is enthalpically driven, and reveal how specific protein-hemin interactions modulate affinity. Notably, other species of gram-positive bacteria mediate hemin uptake using evolutionarily distinct NEAT domains, that despite adopting a protein fold that is distinct from CR domains, nevertheless engage hemin with similar affinity *via* iron-tyrosyl coordination (32, 59). Interestingly, NEAT domains have been shown to transiently associate with one another to rapidly transfer hemin suggesting their placement within the cell wall provides a protein wire that kinetically facilitates hemin movement from the cell surface to the membrane (60, 61). Future studies will need to determine if CR domains are also capable of rapid transfer among one another to facilitate heme movement in Actinobacteria, and define the role of the ChtB and HtaB in the hemin-uptake system that are buried within the cell wall. These results will enhance our understanding of how pathogenic *C. diphtheriae* and other clinically relevant Actinobacteria exploit host Hb as an iron source and may inform the development of novel antibiotics to combat increasingly drug-resistant bacterial pathogens.

## Experimental procedures

### Cloning, expression, and purification of proteins

Initial nucleotide sequencing encoding HtaA, HtaB, and ChtA CR domains were cloned out of *Corynebacterium diphtheria* (NCTC 13129) genomic DNA by PCR using primers flanking the regions encoding HtaA residues 36 to 221 for its N-terminal CR domain (CR1), residues 344 to 506 for its C-terminal CR domain (CR2), HtaB residues 30 to 273 for its CR domain, and ChtA residues 112 to 291 for its CR domain (Integrated DNA Technologies). See Table S5 for list of primer sequences. All expression constructs were verified by Sanger sequencing using the T7 forward and reverse sequencing primers. Alignments were visualized using ENDscript (62). These inserts were cloned into a pET-28b-based plasmid containing an N-terminal small ubiquitin-like modifier (SUMO) fusion tag (pSUMO) and a 6x-His purification tag. Site-directed mutagenesis was used to generate Y178A, Y178H, K177H, Y178H + K177G, Y217M, and Y129A mutants of ChtA^CR^. Resultant plasmids were transformed in *Escherichia coli* BL21 (DE3) chemically competent cells (New England Biolabs) for protein expression. Holo-protein material used for crystallography and ESI-MS was prepared from cultures grown in LB media at 37 °C to a final *A*_600_ of 0.6 to 0.8 before addition of 1 ml of 8 mM hemin chloride dissolved in 0.1 M NaOH and induction with 1 mM isopropyl-β-D-thiogalactopyranoside followed by overnight growth at 17 °C. Cell pellets were harvested by centrifugation and then resuspended in lysis buffer (50 mM sodium phosphate, 300 mM NaCl, pH 7.0) supplemented with 1 mg/ml egg white lysozyme, 2 mM phenylmethylsulfonyl fluoride (Sigma-Aldrich), a protease inhibitor cocktail (Sigma-Aldrich), and 0.5 mg *Serratia marcescens* nuclease (63). Resuspended cells were lysed by sonication and clarified through additional centrifugation. Clarified lysate was added to HisPur Co^2+^-chelating resin (Thermo Fisher Scientific). Nonspecific proteins were washed out with additional lysis buffer supplemented with 10 mM imidazole. Proteins of interest were eluted with high imidazole buffer, dialyzed into low imidazole containing buffer, and the SUMO fusion removed by adding the ULP1 protease (purified in-house). A second passage through a HisPur Co^2+^-chelating resin column was performed to remove the ULP1 from the cleaved protein product. Size-exclusion chromatography was used as a final purification step using an Akta FPLC system (GE Healthcare Life Sciences) equipped with a Superdex S75 size-exclusion column. Analytical size-exclusion chromatography for the ChtA^CR^ domain was performed by injecting 250 μl of protein in 50 mM NaPi, 100 mM NaCl, pH 7.0 onto an Akta FPLC system (GE Healthcare Life Sciences) equipped with an analytical Superdex S75 Increase size. Proteins were concentrated in Amicon Ultra centrifugal filters (EMD Millipore), and final purity was confirmed to be >98% by SDS-PAGE. The apo-form of the CR proteins was obtained by similar methods, but the cells were grown instead in M9 minimal media without any supplementation of exogenous heme. Purification was identical, and UV-visible spectroscopy traces showed no A_405_ Soret peak indicative of bound hemin. H64Y/V68F myoglobin (apo-Mb^H64Y/V68F^) was expressed recombinantly and purified as previously described (64). CR domains that did copurify with some endogenous *E. coli* expression system heme as evidenced by spectral traces had to undergo chemical denaturation and hemin extraction detailed below.

### Preparation of apo and heme bound proteins

CR domain proteins expressed in LB and supplemented with exogenous heme were initially isolated as a mixture of heme-free (apo) and heme-bound (holo) forms. Fully holo proteins were obtained by incubation with 20-fold molar excess hemin chloride dissolved in 0.1 M NaOH while rocking overnight. Separation of excess heme was achieved through the use of diethylaminoethyl (DEAE)-Sepharose anion exchange resin (61), which eliminated the broad free hemin absorbance peak at ∼385 nm observed in spectral traces while maintaining the sharp protein-bound 405 nm Soret band.

CR domains that could not be expressed in the apo-form from *E. coli* were subjected to methyl ethyl ketone followed by removal of the organic layer and refolding of the aqueous protein layer through gentle dialysis back into lysis buffer (65). Removal of bound hemin was confirmed through UV-visible spectroscopy. UV-visible absorbance spectra were collected on a Shimadzu UV-1800 spectrophotometer over the wavelength range 250 to 700 nm in a 1 cm path length cuvette. Proteins were prepared in a buffer of 50 mM sodium phosphate, 100 mM NaCl, pH 7.0.

### Hemin release binding measurements

The hemin release kinetics from metHb were measured by monitoring changes to hemoprotein Soret band absorbance using an established plate reader assay as performed previously (38). Purified metHb, apo-Mb^H64Y/V68F^, apo-ChtA^CR^, apo-HtaA^CR1^, and apo-HtaA^CR2^ were buffer matched in hemin transfer buffer (20 mM sodium phosphate, pH 7.5, 150 mM NaCl, and 450 mM sucrose). Holo-metHb was injected to a final concentration of 5 μM on the heme basis in triplicate with 50 μM of the respective apo protein in a 384-well plate. The absorbance at 405 nm was measured every 2.5 min for 20 h in a SpectraMax iD3 plate reader (Molecular Devices). Experiments were performed at 25 °C to avoid apo-globin aggregation. Resulting curves were fit to a two-phase exponential decay equation in GraphPad Prism version 9.5.0 (GraphPad Software).

### NMR spectroscopy

CR domain proteins were dialyzed against 50 mM sodium phosphate, pH 6.8, 100 mM NaCl. Buffer-matched, blood-purified HbA in the carbonmonoxy form was titrated into the samples to make Hb (heme basis):CR at the following ratios: 1:1, 4:1, 12:1. A BEST-TROSY ^1^H-^15^N heteronuclear single-quantum coherence spectrum was recorded at each titration point. NMR experiments were performed at 298K on a Bruker AVANCE NEO 800 MHz spectrometer equipped with a triple resonance cryogenic probe. NMR spectra were processed using NMRPipe and TopSpin 3.5 (Bruker BioSpin) and analyzed using NMRFAM-SPARKY (66, 67).

### Crystallization, data collection, and structure determination

Purified holo ChtA^CR^ was concentrated to 41.9 mg/ml in 50 mM 2-[4-(2-hydroxyethyl)piperazin-1-yl]ethanesulfonic acid (Hepes), pH 8.0 for broad screening of crystallization conditions. Crystals hits were observed after 1 day at room temperature in a hanging drop, vapor diffusion format against a mother liquor of 0.17 M ammonium sulfate, 25.5% w/v PEG 4000, and 15% v/v glycerol. The crystals were cryoprotected in a solution of mother liquor containing 30% total glycerol for 30 s prior to freezing. Diffraction datasets were collected at Advanced Light Source beamline 8.3.1 equipped with a Dectris Pilatus3 S 6M detector. The crystal was maintained at 90 K during data collection, and diffracted to 1.63 Å resolution. XDS was used to index and integrate reflections (68, 69). The crystal belongs to space group C222_1_, and has a Matthews coefficient of 2.59 Å^3^/Da and a 52.5% solvent content (70, 71, 72). The data were moderately anisotropic so additional ellipsoidal processing and anisotropic scaling was performed using the STARANISO server (73). An off-origin peak in the Patterson map was observed at fractional coordinates (0.5, −0.053, 0.5) with a peak height of 53.528% relative to the origin and was present in all datasets for the condition, suggestive of translational noncrystallographic symmetry. This peak is reproduced in a Patterson map derived from the calculated intensities of the atomic model, ruling out the possibility of a translocation disorder (74), and given the high quality of diffraction data, a meaningful structure solution could still be obtained (75, 76, 77). PHASER was used for molecular replacement using an Alphafold2 prediction of the ChtA^CR^ as a search model from the AlphaFold Protein Structure Database (AFDB ID: AF-Q6NGJ3-F1, Fig. S10), which led to a single solution (LLG: 4226.318) (78, 79). Model building proceeded using Coot and refinement at a resolution limit cutoff of 1.63 Å proceeded using BUSTER using LSSR-type restraints for NCS and TLS for the individual domains (80, 81, 82, 83). Statistics for data collection and refinement are shown in Table S2, with final coordinates and structure factors deposited under Protein Data Bank (PDB) code: 9O0K.

Purified holo HtaA^CR1^ was concentrated to 10.1 mg/ml in 50 mM Hepes, pH 8.0 for broad screening of crystallization conditions. Crystals hits were observed after 4 days at room temperature in a hanging drop, vapor diffusion format against a mother liquor of 0.2 M lithium sulfate, 0.1 M sodium acetate, pH 4.5, and 50% PEG 400. Due to the high PEG concentration of the mother liquor, crystals were not additionally cryoprotected with glycerol before freezing, and no ice rings were observed in subsequent diffraction data. Diffraction datasets were collected at Advanced Light Source beamline 8.3.1 equipped with a Dectris Pilatus3 S 6M detector. The crystal was maintained at 90 K during data collection, and diffracted to 1.88 Å resolution. XDS was initially used to index and integrate reflections (68, 69). The apparent space group was C222, resulting in a Matthews coefficient of 2.14 Å^3^/Da and a 42.6% solvent content (70, 71, 72). The data were extremely anisotropic so additional ellipsoidal processing and anisotropic scaling was performed using the STARANISO server (73). PHASER was only able to place four copies of an Alphafold2 search model from the AlphaFold Protein Structure Database (AFDB ID: AF-Q6NIZ1-F1, Fig. S10) in the ASU out of the expected 10 based on the Matthews coefficient (LLG: 314), but attempts to model in a fifth copy based on empty space and residual density analysis caused a symmetry clash (78, 79). Xtriage analysis suggested unusual intensity statistics indicative of twinning, with the Yeates-Padilla L-test strongly suggesting a near perfect twin (α = 0.46) (84, 85). As twinning was not permitted in the indexed space group, molecular replacement proceeded in the lower symmetry space group of C121, which corresponded to a predicted Matthews coefficient of 2.14 Å^3^/Da and a 42.6% solvent content for 20 molecules in the ASU. A single solution placing 10 copies in the ASU was obtained, with no additional copies appearing to be missing upon inspection of residual density (LLG: 744), corresponding to a Matthews coefficient of 4.28 Å^3^/Da. Despite the high solvent content (71.3%), the molecules in the ASU now formed a β-laddering pattern that bridged between adjacent unit cells, likely stabilizing the crystal lattice. This initial solution underwent iterative manual modeling building in Coot and refinement using Phenix.refine with NCS restraints, TLS for the individual domains, and the twin law setting enabled (twin operator = h, -k, -l; twin fraction = 0.5) (80, 86, 87, 88). Statistics for data collection and refinement are shown in Table S2, with final coordinates and structure factors deposited under Protein Data Bank code: 9O0J.

### Resonance Raman spectroscopy

Protein samples were concentrated to 0.5 mM in a phosphate-buffered solution (50 mM NaPi, pH 7.0, 100 mM NaCl) and placed in Suprasil EPR sample tubes and frozen with liquid nitrogen (Wilmad). Samples were maintained frozen in a 50 ml Suprasil symmetric nitrogen dewar flask during data collection (Wilmad). The resonance Raman setup was similar to that described previously (89, 89). Laser excitation wavelengths of 457 nm and 488 nm were generated by a Cobolt 08-01 diode pumped laser series equipped in a C-Flex C6 laser combiner (HÜBNER Photonics, Kassel). The spectra obtained with 407 nm excitation employed a tunable titanium:sapphire laser (Spectra-Physics Tsunami) pumped by a 15 W diode pumped solid state laser (Spectra-Physics Millennia eV) and configured with a 10 ps Gires−Tournois interferometer. The ∼4.0 W fundamental beam at 814 nm was focused into a β-barium borate crystal (Eksma Optics) for second-harmonic generation, and the ∼3 mW of 407 nm light was separated from the fundamental with a Pellin−Broca prism and a dichroic mirror. The beam was focused onto the sample at 135° backscattering angle using an f/4 parabolic focusing mirror. The scattered light was collimated by an f/0.8 UV-fused silica aspheric lens (Edmund Optics), focused onto the 100 μm slit of an f/4.6 single-grating spectrograph equipped with an 1800 gr/mm grating blazed at 500 nm (Princeton Instruments Isoplane 320) and imaged onto a Peltier-cooled CCD (Princeton Instruments Pixis 100B). Rayleigh scattering was rejected by appropriate edge filters for each wavelength (Semrock, RazorEdge). A 50/50 (% v/v) mixture of toluene and acetonitrile was used to calibrate the spectrograph to within ± 1 cm^−1^. Laser power at the samples was set to 1.4, 12, or 10 mW for 407 nm, 457 nm, and 488 nm, respectively. An individual spectrum was collected in 1 min increments over a 60 to 120 min time period. Spectra were exported and analyzed in Igor Pro 9 (WaveMetrics). Measured spectra were summed to a final spectrum after manually removing cosmic rays impinging on the detector over data collection. Spectral features from the buffer solution and quartz were then subtracted from the summed spectrum. The resulting spectrum was baseline-corrected to account for any intrinsic fluorescence or scatter off the sample holder. The baseline-corrected spectrum was normalized to the most intense peak in each spectrum.

### EPR spectroscopy

Continuous-wave X-band EPR spectra were collected at 5 K using a Bruker EMXplus instrument equipped with an Oxford Instruments ESR 900 continuous flow helium cryostat and a MercuryiTC temperature controller (Oxfordshire) in the Molecular Instrumentation Center at University of California, Los Angeles. All EPR spectra were recorded using a microwave power of 20 mW, microwave frequency of 9.36 GHz, modulation frequency of 100 kHz, and modulation amplitude of 8 G. Background signals from phosphate buffer solution were removed by subtracting the buffer spectrum in Igor Pro 9.00 (Wavemetrics).

### Isothermal titration calorimetry

Thermodynamic parameters for the interaction between hemin and the ChtA^CR^ domain and mutants were measured using an ITC200 (GE Healthcare). ∼3.5 mg of hemin chloride was dissolved in 5 ml of dimethyl sulfoxide (DMSO). Immediately before each titration, a 500 μL aliquot of the heme solution in DMSO was diluted into 9.5 ml of titration buffer (50 mM Tris–HCl, pH 8.0, 200 mM NaCl) and rigorous quantification of the working sample by UV-visible spectroscopy was carried out (ε_0_ = 170,000 mM^−1^ cm^−1^ for hemin in 80–100% DMSO (90), working hemin stock diluted 1:10 in pure DMSO for measurement). Protein samples were equilibrated in titration buffer supplemented with 5% (v/v) DMSO. Aliquots containing ChtA and mutants (300–500 μM) were injected stepwise into the cell of the calorimeter containing the hemin (25–33 μM) solution. Binding isotherms were fit to a one-site binding model using the program ORIGIN (91).

### Native ESI-MS

nESI-MS was used for ligand (heme)-competition experiments between CR domain pairs to determine their relative heme dissociation constant K_relative_ (K_rel_) (37, 56). In this approach, two different CR domains (*e.g.* a >90% heme-loaded “heme donor” and a <10% heme-loaded “heme acceptor” CR domain) are mixed and allowed to come to equilibrium (>6 h, 24 °C). The resulting mixture is then analyzed by nESI-MS, where the apo- and holo-abundance of each CR domain is determined from the sum peak intensity from each of their charge states as described previously (37). The relative affinity between the CR pair tested can be determined by their nESI-MS abundances according to Equation 1, which is derived from the ratio of each CR’s K_D_ for heme. Importantly, this analysis is independent of [heme] and K_rel_ should be consistent across different concentrations of [heme donor] and [heme acceptor]; thus, we used at least three different holo-donor and apo-acceptor stoichiometry ratios to determine each K_rel_ reported in Table S4.

(eq. 1) $$K_{Relative}=\frac{\left[{CR}^{heme\;acceptor}\right]\;\left[{CR}^{heme\;donor}\cdot heme\right]}{\left[{CR}^{heme\;donor}\right]\;\left[{CR}^{heme\;acceptor}\cdot heme\right]}=\frac{Ab\left({CR}^{heme\;acceptor}\right)\;Ab\left({CR}^{heme\;donor}\cdot heme\right)}{Ab\left({CR}^{heme\;donor}\right)\;Ab\left({CR}^{heme\;acceptor}\cdot heme\right)}$$

For nESI-MS heme-competition experiments, CR domains were first buffer-exchanged into 50 mM ammonium acetate (pH 6.9) using dialysis or gel filtration and protein concentration was determined by bicinchoninic acid assay or UV-visible absorbance at 280 nm. Proteins were then mixed at donor:acceptor ratios ranging from 1:9 to 9:1 (but most often 1:3, 1:1, and 3:1), and incubated for at least 6 h at 24 °C. Equilibrated mixtures were then loaded into platinum-coated borosilicate emitter tips with an inner diameter of 200 to 500 nm pulled on a Sutter P-1000 Micropipette Puller and sprayed on a Q-Exactive UHMR Hybrid Quadrupole-Orbitrap Mass Spectrometer (Thermo Fisher Scientific).

## Data availability

The structures of ChtA^CR^:hemin and HtaA^CR1^:hemin have been deposited in the PDB under accession numbers 9O0K and 9O0J, respectively. Coordinates for HtaA^CR2^:hemin can be found under accession number 8SMU.

## Supporting information

This article contains supporting information (29, 45, 46, 49, 50, 53, 92, 93).

## Conflict of interest

The authors declare that they have no conflicts of interest with the contents of this article.
