# Supplementary material for: Structural basis of heme scavenging by the ChtA and HtaA hemophores in Corynebacterium diphtheriae
Source: J Biol Chem. 2025 Aug 26;301(10):110633. doi: 10.1016/j.jbc.2025.110633 (PMC12494546; doi:10.1016/j.jbc.2025.110633)
Supplement: Supporting Figures and Tables [file mmc1.docx]

**SUPPLEMENTAL MATERIAL:**

**Structural Basis of Heme Scavenging by the ChtA and HtaA Hemophores in *Corynebacterium diphtheriae***

Jordan Ford^1^, Andrew K. Goring^1^, Yuri Lee^1^, Megan Chen^1^, Brendan J. Mahoney^1,2^, Michael R. Sawaya^2^, Hannah S. Shafaat^1^, Joseph A. Loo^1,2,3^, and Robert T. Clubb^1,2,3^*

^1^Department of Chemistry and Biochemistry, University of California, Los Angeles, 611 Charles E. Young Drive East, Los Angeles, CA 90095, USA.

^2^UCLA-DOE Institute of Genomics and Proteomics, University of California, Los Angeles, 611 Charles E. Young Drive East, Los Angeles, CA 90095, USA.

^3^Molecular Biology Institute, University of California, Los Angeles, 611 Charles E. Young Drive East, Los Angeles, CA 90095, USA.

**This file includes:**

Supplementary Text

Figures S1 to S10

Tables S1 to S5

SI References

**
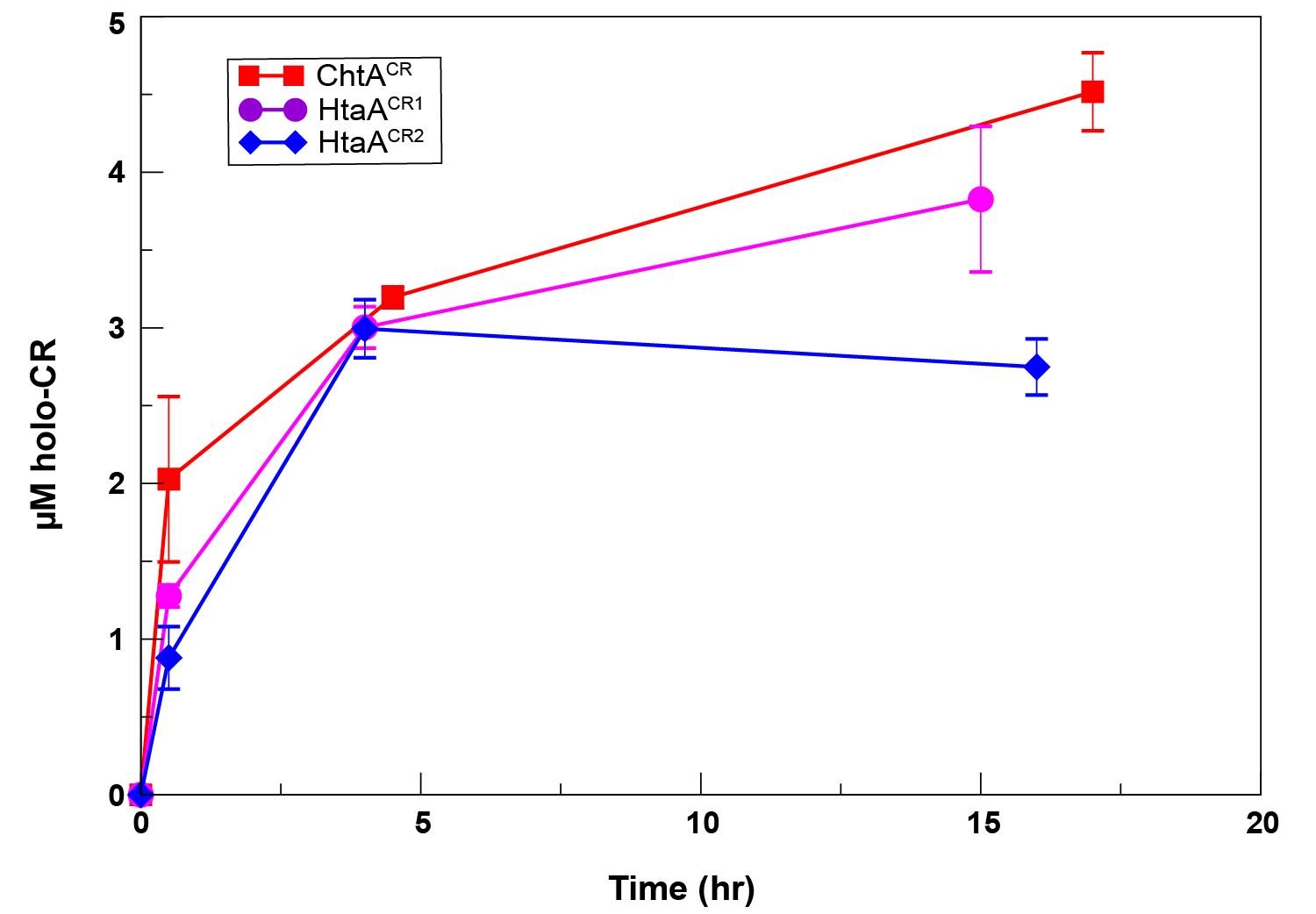
**

**Figure S1.** **CR domains passively capture hemin released from Hb by mass spectrometry.** In separate experiments CR domains (ChtA^CR^, HtaA^CR1^ or HtaA^CR2^) were incubated with 5 µM metHb and the degree of hemin transfer determined by native mass spectrometry. When metHb is mixed with a 4-fold molar excess of the apo-form of each CR domain hemin transfer is observed. The kinetics of this process are similar to those measured by UV-Vis spectroscopy (**Fig. 2**) and are compatible with metHb’s hemin molecules first being released into the solvent before being captured by the CR domains. Error bars represent the standard deviation of three measurements.


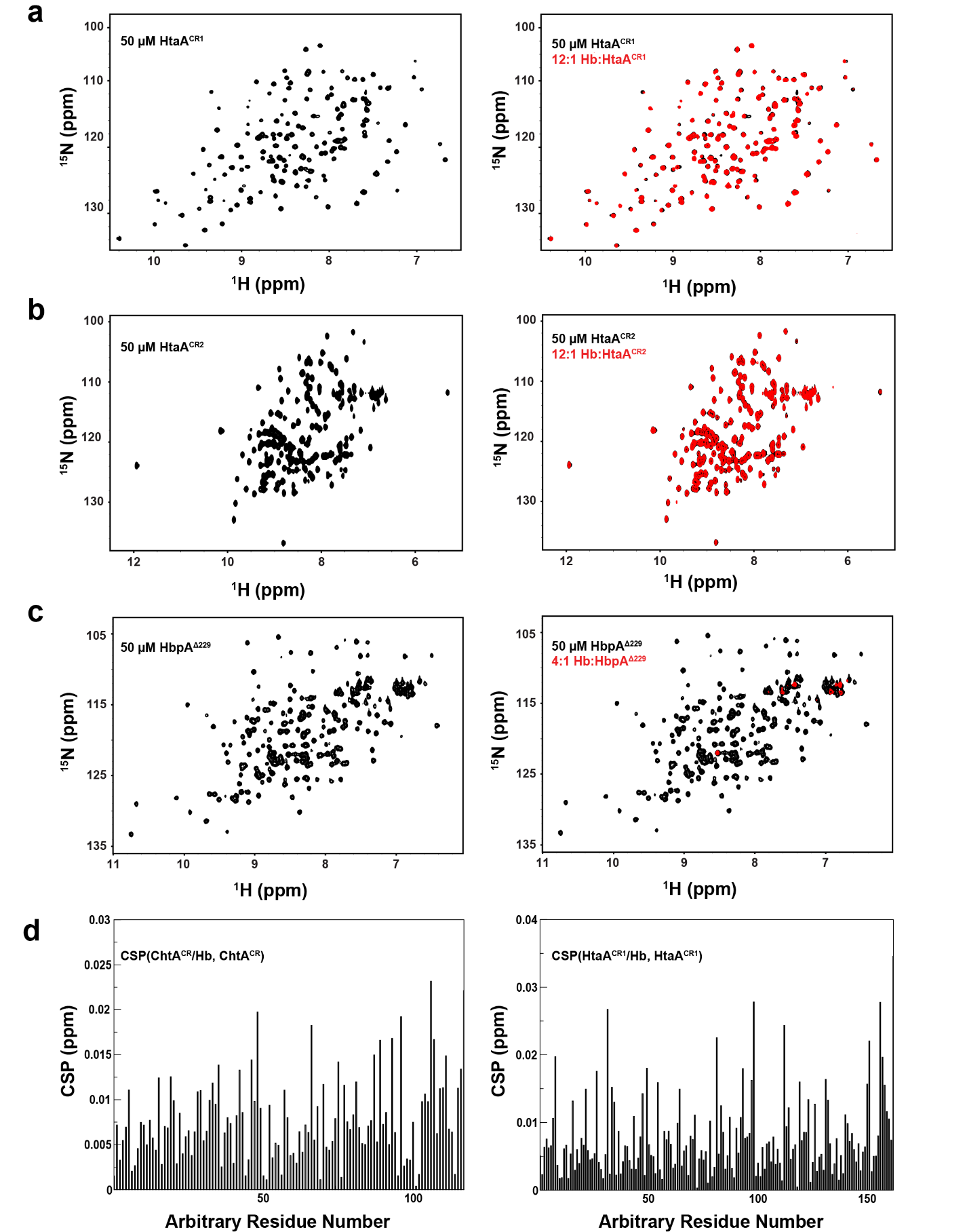


**Figure S2. NMR spectral comparison of CR domains from HtaA and the HbpA receptor protein binding to hemoglobin.** (a) ^1^H-^15^N HSQC spectrum of ^15^N-labeled apo-HtaA^CR1^ alone (left) and overlaid with the spectrum when 12-fold molar excess of unlabeled Hb (heme basis) is present. No significant spectral changes are observed after the addition of Hb indicating that the proteins do not interact with one another with appreciable affinity. (b) Same as in (a) but for previously characterized apo-HtaA^CR2 1^. (c) ^1^H-^15^N HSQC spectra of ^15^N-labeled HbpA receptor protein from *Corynebacterium diphtheriae* alone (left) and overlaid with the spectra when 4-fold molar excess of unlabeled Hb (heme basis) is present^1^. Even at a significantly lower molar excess of Hb, the majority of peaks disappear in the complex spectra, indicative of a higher molecular weight species forming. (d) Arbitrary chemical shift perturbation (CSP) plots for lone ChtA^CR^ versus ChtA^CR^ in the presence of 12-fold molar excess Hb (left) or lone HtaA^CR1^ versus HtaA^CR1^ in the presence of 12-fold molar excess Hb. Peak positioning is not significantly perturbed in the Hb containing spectra. In all cases, the carbonmonoxy form of Hb was used, which does not release heme to a significant extent during the time course of the experiment.


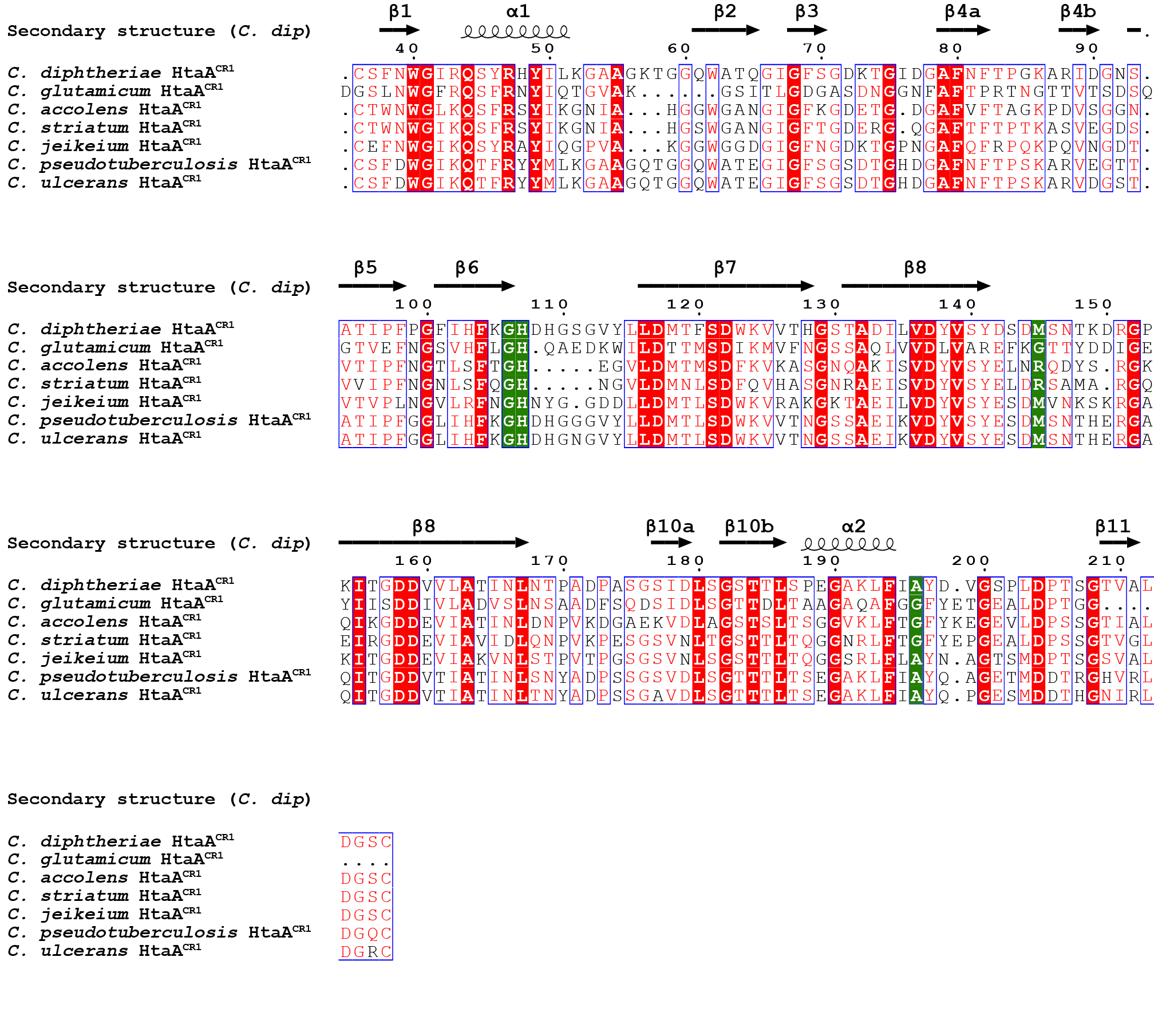


**Figure S3. Secondary structure map and alignment of HtaA^CR1^ domains across different *Corynebacteria* species.** Alignment and secondary structure annotation based on the crystal structure of the *Corynebacterium diphtheriae* HtaA^CR1^ domain in a variety of Corynebacteria species. The axial-adjacent G106 & H107 are strongly conserved features across all hitherto observed CR domains, with the exception of the ChtA-CR type domain reported herein. Though the identity of the capping A196 is always a small residue (G or A), the capping M145 observed in *Corynebacterium diphtheriae* is conserved only in the obligate pathogens (*C. diphtheriae, C. jeikeium, C. ulcerans, C. pseudotuberculosis*) and not the commensals/soil bacteria (*C. accolens, C. striatum, C. glutamicum,* respectively).


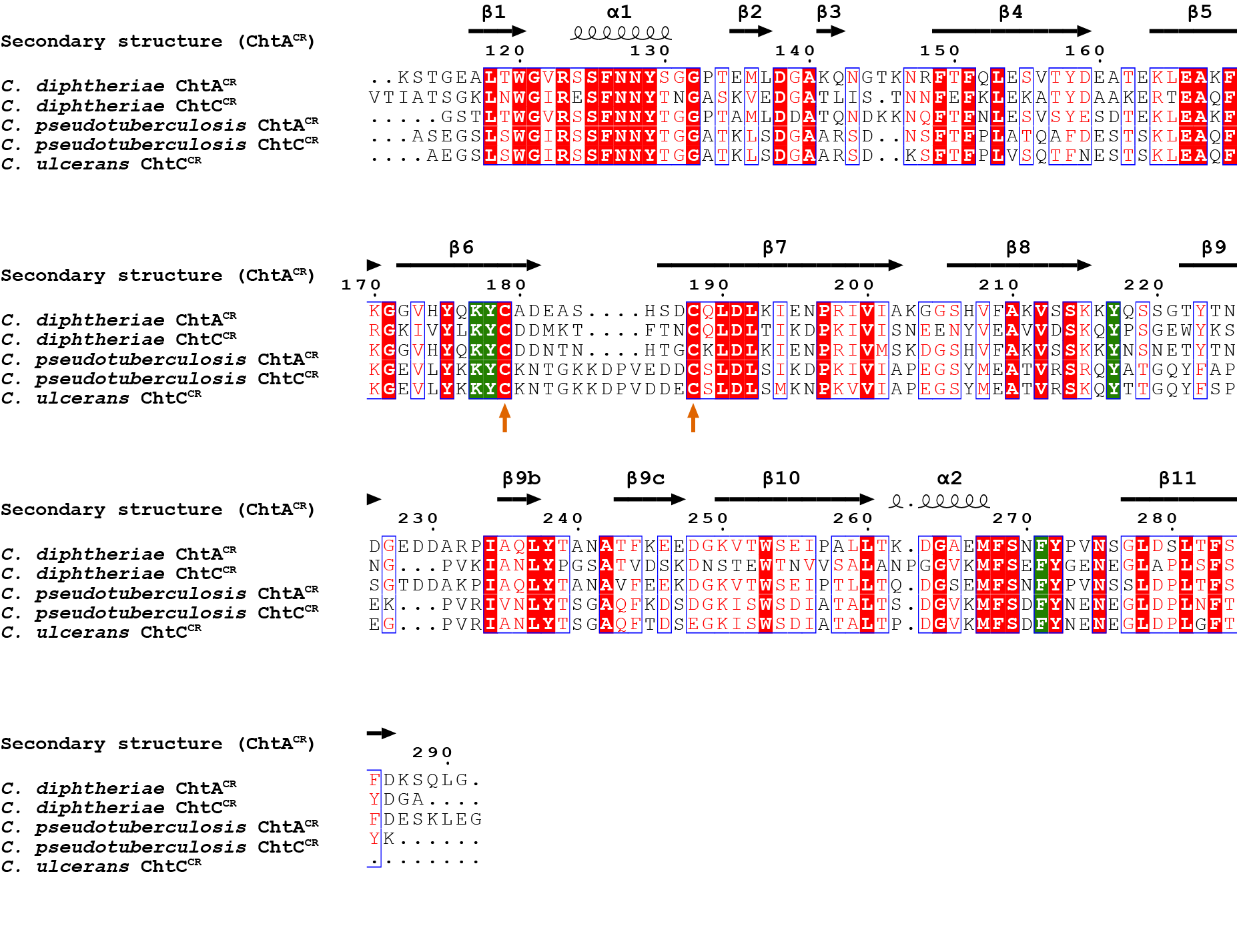
**Figure S4. Secondary structure map and alignment of ChtA/C-type CR domains across different *Corynebacteria* species.** Alignment and secondary structure annotation based on the crystal structure of the *Corynebacterium diphtheriae* ChtA^CR^ domain in pathogenic Corynebacteria species. Many of the observed novel heme pocket features, such as the capping residues Y217 & F271 and axial-helper residue pair K177 & Y178 (green boxes) and the disulfide-gated β6–β7 loop insertion (C179-C188, orange arrows), are shown to be strongly conserved and diagnostic of this particular type of CR domain.


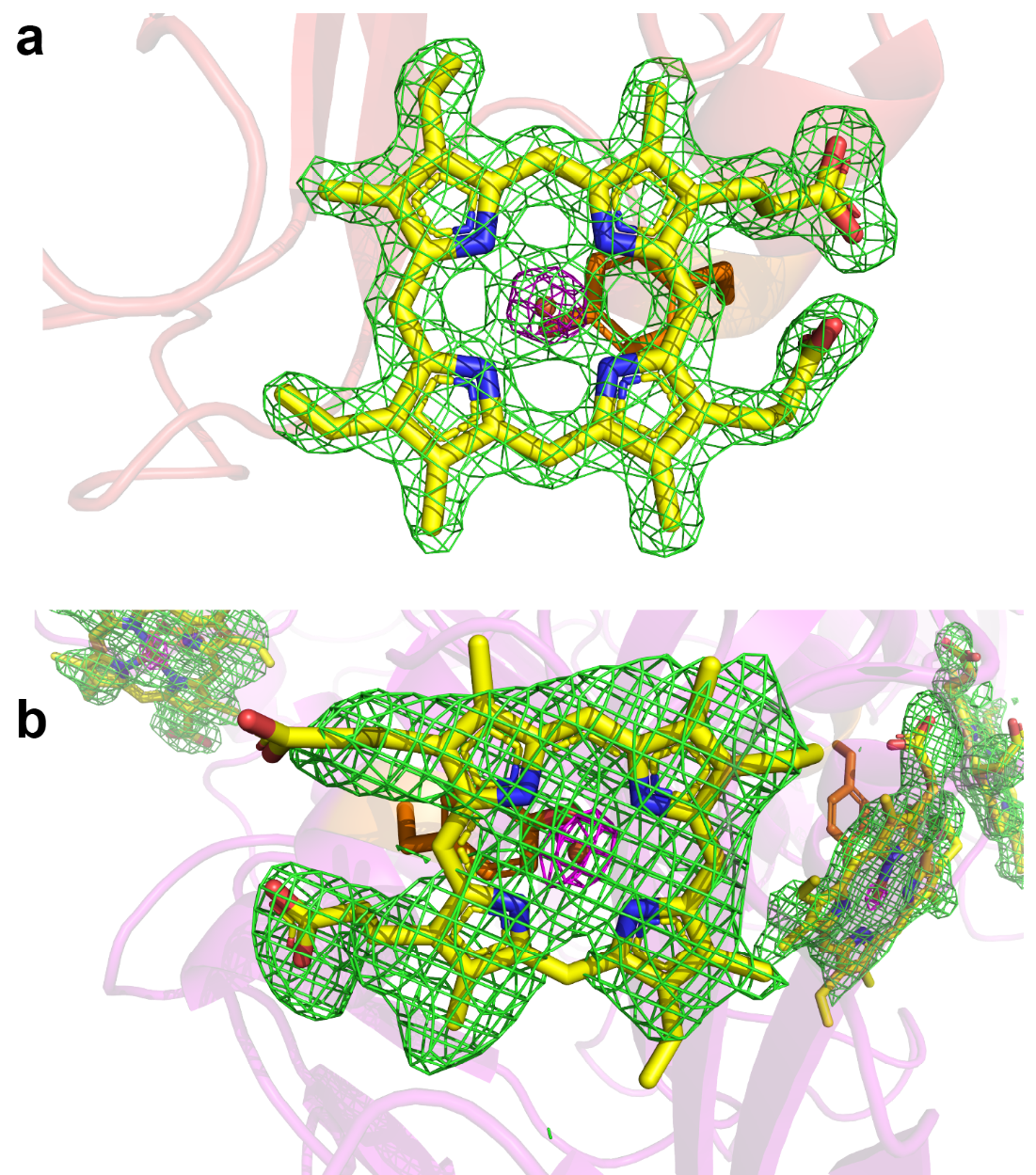


**Figure S5. Omit maps for bound HEM ligand.** (a) Unbiased omit map depicting Fe(III)-PPIX (heme) bound to ChtA^CR^. The sigma-A weighted omit Fo − Fc electron density map is contoured at 4.0 σ (green) and 20 σ (purple). (b) Unbiased omit map depicting Fe(III)-PPIX (heme) bound to HtaA^CR1^. The sigma-A weighted omit Fo − Fc electron density map is contoured at 3.0 σ (green) and 10 σ (purple). Carbon atoms of porphyrin and protein are depicted in yellow and orange, respectively. Oxygen atoms are shown in red, and nitrogen atoms in blue. The purple sphere corresponds to Fe(III).


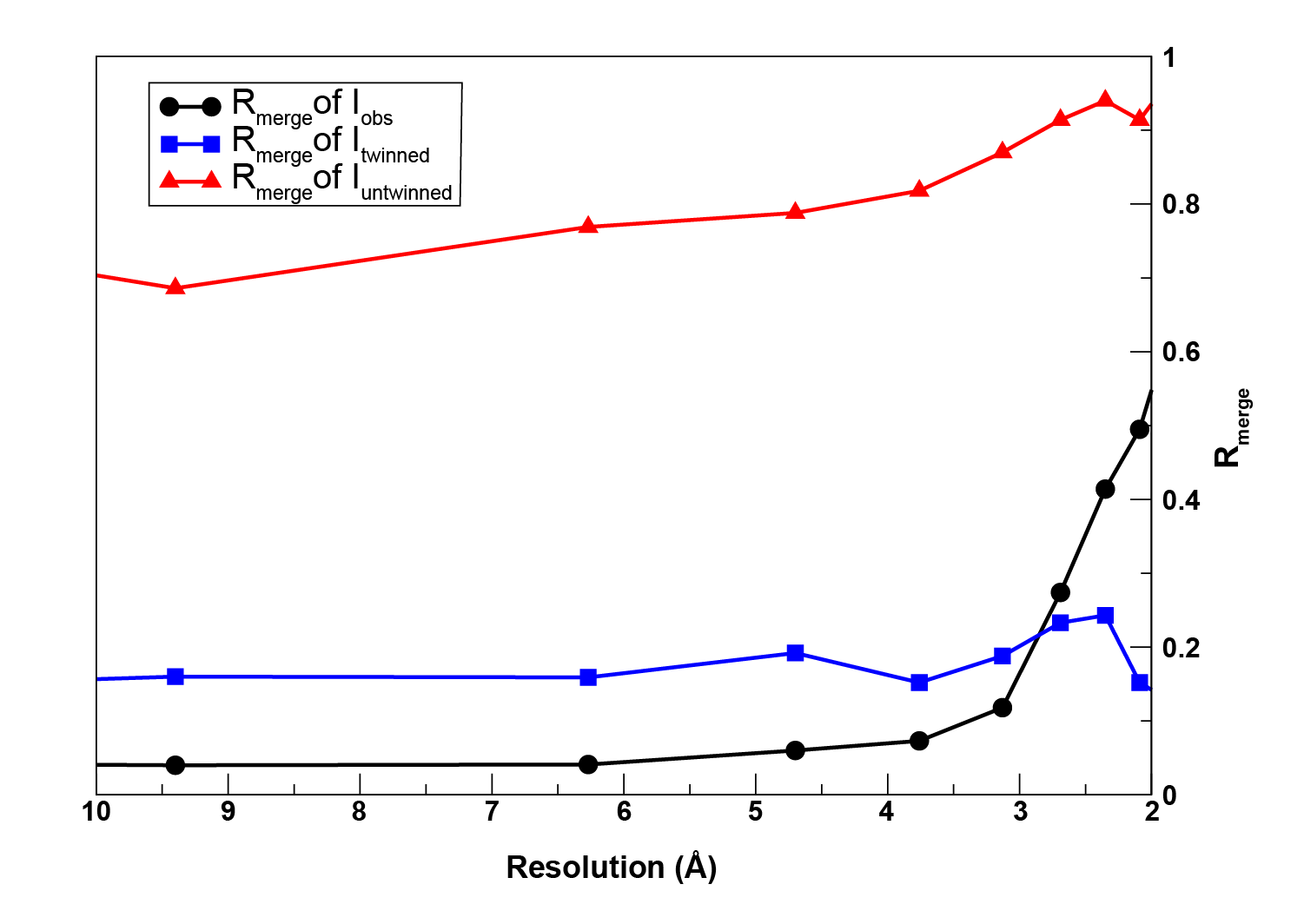


**Figure S6.** **Comparison of model quality with twin law implementation in C121.** Comparison of R_merge_ values versus resolution shells for twin-related reflections in space group C121 for the structure of HtaA^CR1^, shown for R_merge_ values on I_calc_ without twin law implementation (red triangles), on *I_calc_* with twin law implementation (blue squares) and *I_obs_* (black circles). While R_merge_ on *I_obs_* is approximately 10% across the resolution range, R_merge_ on *I_calc_* before implementation of the twinning operator is approximately 85%. Implementation of the twinning operator brings R_merge_ on *I_calc_* down to a reasonable threshold. The improved ability to model the close agreement between twin-related intensities by use of the twin operator provides strong evidence for the presence of twinning.


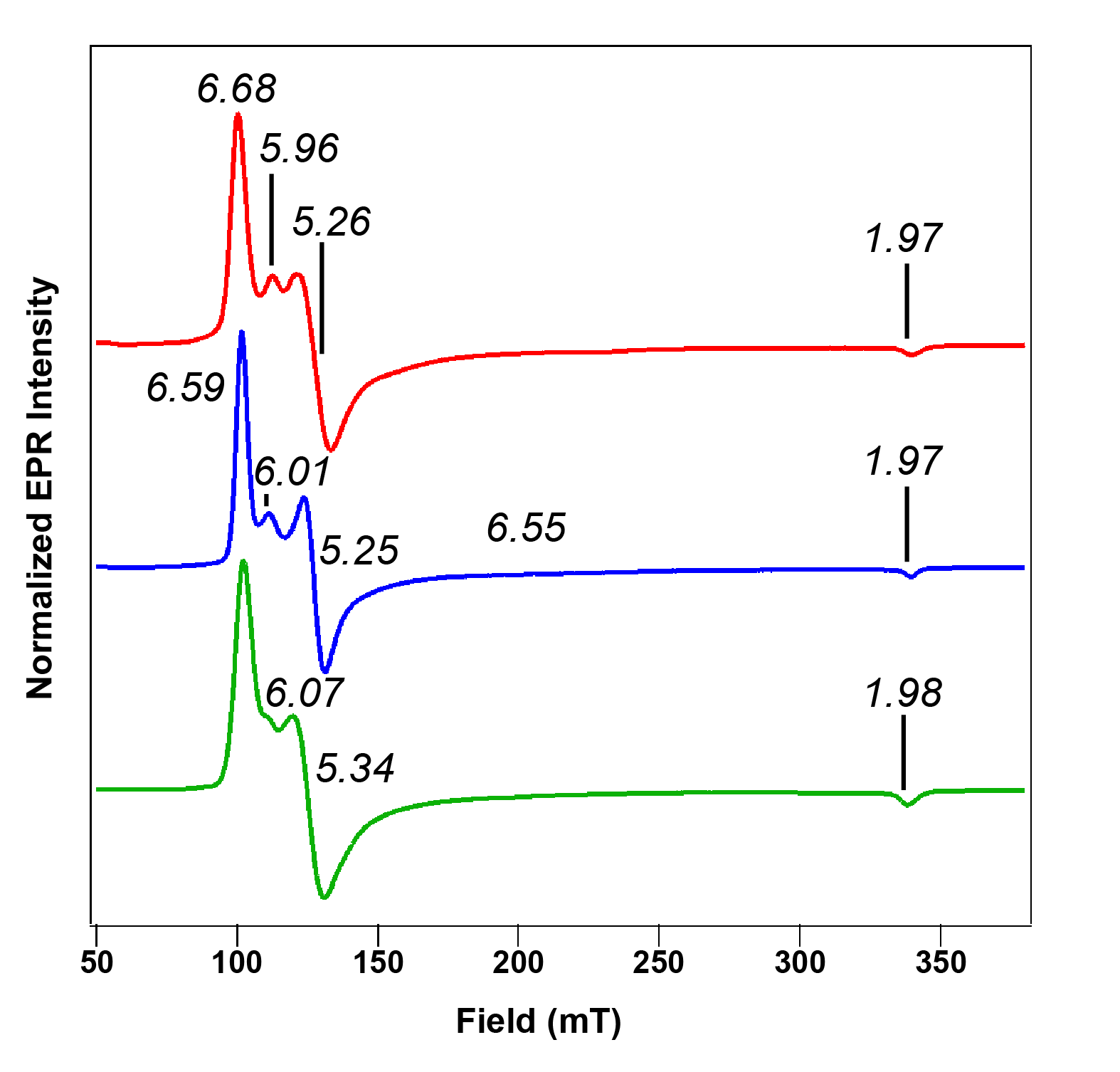


**Figure S7.** **EPR spectra of hemophore CR domains.** X-band EPR spectra of ChtA^CR^ (red), HtaA^CR1^ (blue), and HtaA^CR2^ (green) with apparent *g*-values indicated. Experimental conditions: microwave frequency 9.36 GHz, microwave power 20 mW, modulation amplitude 8 G, modulation frequency 100 kHz, conversion time 41.60 ms, time constant 40.96 ms, and temperature 5 K.


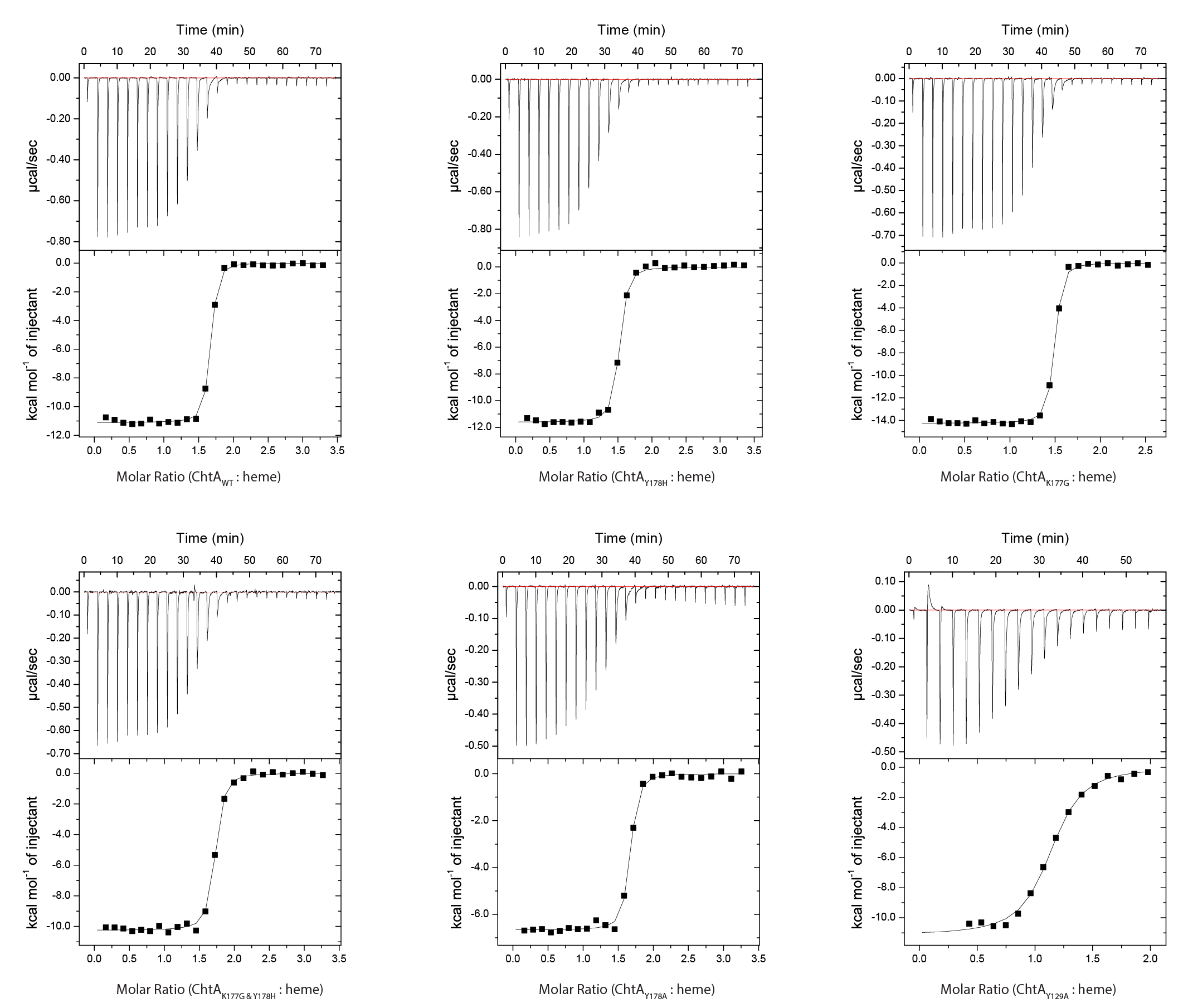


**Figure S8. ITC data and fitted integrated isotherms for titration of hemin with ChtA^CR^ variants.** Representative Isothermal Titration Calorimetry (ITC) data showing hemin binding to ChtA^CR^ and mutants listed in Table 1. The binding isotherm was obtained by injecting apo-protein (500 μM) into a cell containing hemin chloride (30 μM) at a constant temperature of 25 °C. (top) Time-course of the titration (black) and baseline (red). (bottom) Fitted integration of the isotherm. Thermodynamic parameters were obtained by non-linear regression to a one-site binding model with the program ORIGIN.


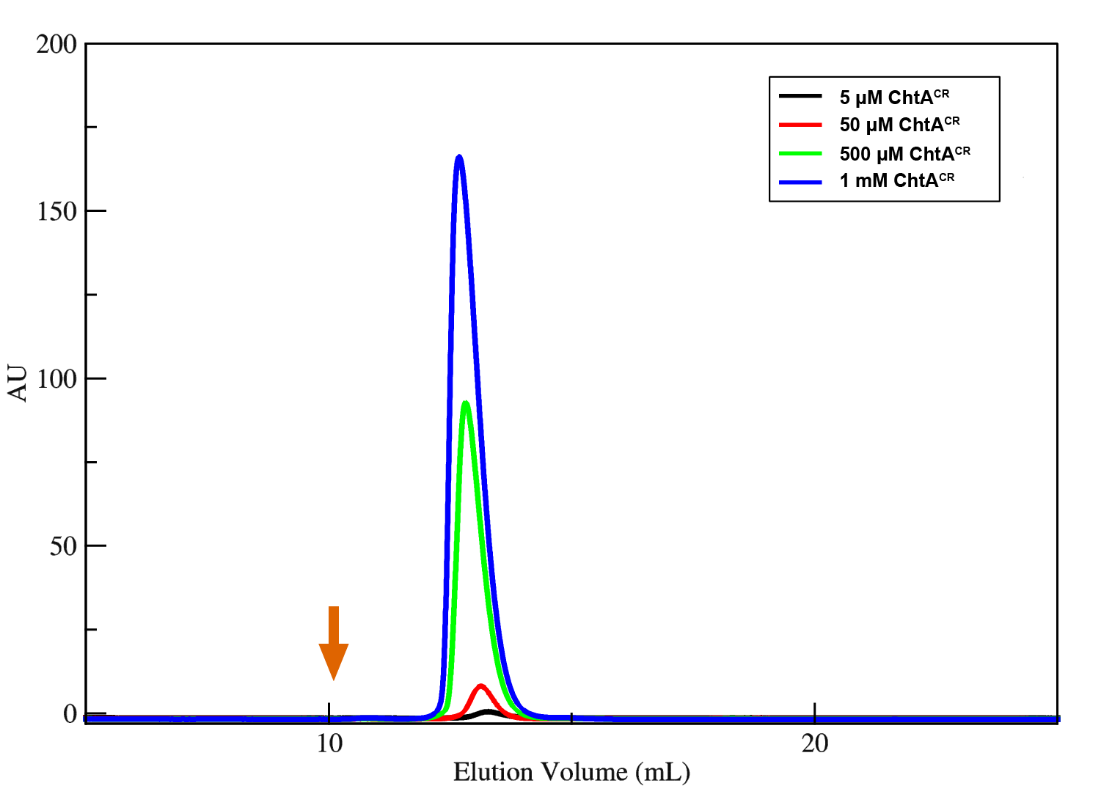


**Figure S9. Analytical gel filtration suggests apo ChtA^CR^ remains monomeric.** Chromatogram overlay of >90% apo ChtA^CR^ domain at differing concentrations that encompass and surpass conditions in the ITC cell or syringe (~5-80 μM or ~500 μM, respectively). The lack of any significant peak shifts or the appearance of a higher molecular weight species earlier in the elution volume suggests the protein remains monomeric in solution, eluting between ribonuclease A (13.7 kDa) and chymotrypsinogen A (25.6 kDa). The expected monomeric kDa of ChtA^CR^ is 19.8 kDa. The orange arrow shows the expected elution volume for a 39.6 kDa dimer (trace for low molecular weight standards not shown).


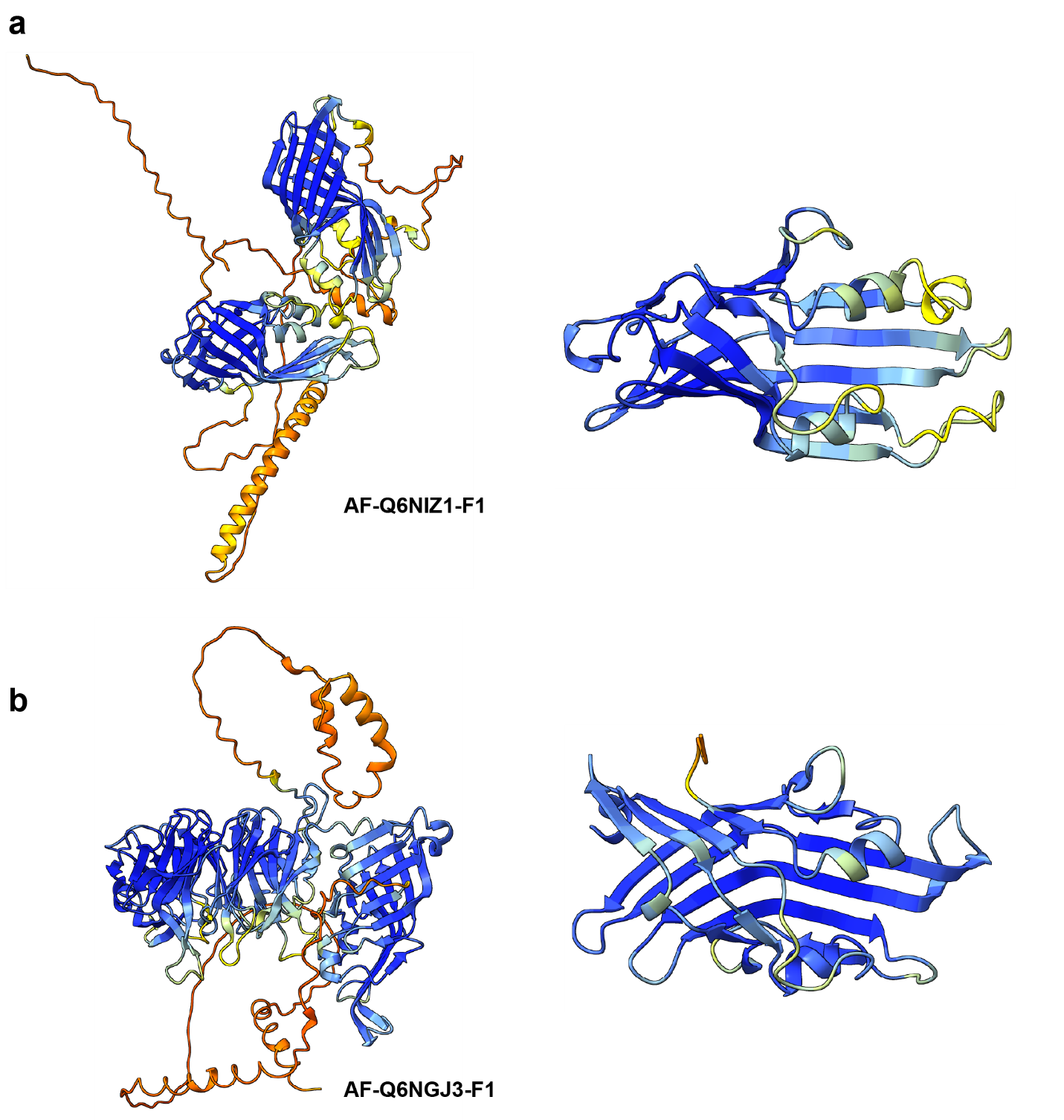


**Figure S10. Alphafold molecular replacement search models for structure determination.** Alphafold2 models from the Alphafold protein database for predicted structures used as molecular replacement search models during phasing. (a) Model for *Corynebacterium diphtheriae* HtaA (AFDB ID: AF-Q6NIZ1-F1) full-length protein (left) and fragment used for HtaA^CR1^ search model (right). (b) Model for *Corynebacterium diphtheriae* ChtA (AFDB ID: AF-Q6NGJ3-F1) full-length protein (left) and fragment used for ChtA^CR^ search model (right). Color represents Alphafold2 pLDDT confidence score, where: Dark blue, very high confidence (pLDDT > 90); teal, high confidence (90 > pLDDT > 70); yellow, low confidence (70 > pLDDT > 50); and orange, very low confidence (pLDDT < 50).

**Table S1.** Kinetic parameters for the rate of hemin dissociation from metHb in the presence of apo CR domains

| **Apo-acceptor** | ***k*_fast_ (hr^-1^)** | ***k*_slow_ (hr^-1^)** | **%*k*_fast_** | ***k_fast_/k_slow_*** |
| --- | --- | --- | --- | --- |
| Myoglobin^H64Y/V68F^ | 1.05 [0.85 - 1.31] | 0.13 [0.11 - 0.14] | 34.6% [30.8% - 38.3%] | 8.09 |
| ChtA^CR^ | 1.78 [1.51 - 2.1] | 0.18 [0.17 - 0.19] | 35.52% [33.1% - 37.9%] | 9.67 |
| HtaA^CR1^ | 1.55 [1.13 - 2.15] | 0.21 [0.18 - 0.23] | 33.11% [26.9% - 39%] | 7.55 |
| HtaA^CR2^ | 2.46 [2.23 - 2.72] | 0.21 [0.19 to 0.22] | 41.11% [39.3% - 42.9%] | 11.95 |

Kinetic parameters characterizing the loss of hemin from metHb in the presence of apo-acceptor. Fits were obtained by monitoring the reduction in A_405_ after injecting 5 µM metHb on a hemin basis into 50 µM apo-protein. Reactions were best fit to a biphasic exponential decay equation. Bracketed values represent the 95% confidence interval for the fitted parameter values.

**Table S2.** Structural statistics for the ChtA^CR^-hemin and HtaA^CR1^-hemin complexes

|  | **ChtA^CR^-Hemin** | **HtaA^CR1^-Hemin** |
| --- | --- | --- |
| **Data collection** |  |  |
| Space group | C 2 2 2_1_ | C 1 2 1 |
| Cell dimensions |  |  |
| *a*, *b*, *c* (Å) | 37.3, 188.8, 119.2 | 106.7, 209.1, 153.9 |
| α, β, γ (°) | 90.0, 90.0, 90.0 | 90.0, 90.0, 90.0 |
| Resolution (Å) | 19.44-1.63 (1.80-1.63) | 19.94-1.88 (2.09-1.88) |
| Wavelength (Å) | 1.115830 | 1.115790 |
| Total observations | 408168 (9317) | 792670 (43990) |
| Unique reflections | 34917 (1745) | 147873 (7394) |
| *R_merge_* (%)  R_pim_ (%) | 0.122 (0.997)  0.037 (0.451) | 0.103 (1.028)  0.048 (0.458) |
| *I* / σ*I* | 10.3 (1.68) | 9.6 (1.66) |
| CC_1/2_ | 0.997 (0.585) | 0.997 (0.474) |
| Completeness (%) | 89.0 (47.6)* | 92.9 (71.0)* |
| Multiplicity | 11.7 (5.3) | 5.4 (5.9) |
| Wilson B-factor (Å^2^) | 16.6 | 24.2 |
| **Refinement** |  |  |
| Resolution (Å) | 17.47 - 1.63 | 19.94-1.88 |
| No. of reflections | 34869 | 147859 |
| *R_work_* / *R_free_* (%) | 23.1/25.6 | 21.65/24.82 |
| No. atoms | 3127 | 14133 |
| Protein | 2740 | 13249 |
| Ligand/ion | 163 | 502 |
| Water | 224 | 382 |
| *B*-factors (Å^2^) (all atoms) | 24.8 | 37.4 |
| Protein | 23.6 | 38.9 |
| Ligand/ion | 32.0 | 33.3 |
| Water | 34.6 | 25.7 |
| R.m.s. deviations |  |  |
| Bond lengths (Å) | 0.01 | 0.01 |
| Bond angles (°) | 1.62 | 1.25 |
| Ramachandran favored (%) | 98.85 | 96.02 |
| Ramachandran allowed (%) | 0.57 | 3.41 |
| Ramachandran outliers (%) | 0.57 | 0.57 |
| PDB ID | 9O0K | 9O0J |

Statistics for the highest resolution shell are shown in parentheses.

*Values for completeness are reported for the ellipsoidal shells. Spherical completeness values for ChtA^CR^ are 65.6 overall, with 100 for the lowest resolution shell and 13.1 for the highest resolution shell. Spherical completeness values for HtaA^CR1^ are 54.6 overall, with 99.8 for the lowest resolution shell and 10.3 for the highest resolution shell.

**Table S3**. Resonance Raman frequencies and intensities for select previously reported heme-tyrosinate proteins.

| **Protein** | **Coordination number and spin state** | | **ν_2_ (cm^-1^)** | **ν_3_ (cm^-1^)** | **ν_4_ (cm^-1^)** | **ν_Fe-O_ (cm^-1^)** | **ν_10_ ν_C=C, vinyl_ (cm^-1^)** | ***I_ν3_/I_ν4_*** | **Ref.** |
| --- | --- | --- | --- | --- | --- | --- | --- | --- | --- |
| ChtA^CR^ | | 5c HS | 1573 | 1496 | 1377 | 584 | 1632 | 1.3 | This work |
| HtaA^CR1^ | | 5c HS | 1567 | 1488 | 1374 | 587 | 1625 | 6.2 | This work |
| HtaA^CR2^ | | 5c HS | 1568 | 1494 | 1374 | 590 | 1628 | 2.3 | This work |
| HmbR | | 5c HS | 1570 | 1489 | 1370 | 518 | 1622 (*v*(C=C)_vinyl_) and 1629 (*v*_10_) | 1.4 | ^2^ |
| ShuT | | 5c HS | 1565 | 1484 | 1369 | 613 | 1616 (*v*_10_) | 1.2 | ^3^ |
| hHO-1(H25Y) | | 5c HS | 1570 | 1490 | 1371 | 591 | 1625 (*v*_10_) | - | ^4^ |
| Mb(H93Y) | | 5c HS | 1572 | 1492 | 1372 | 585 | 1626 (*v*_10_) | - | ^5^ |
| Hb M Boston | | 5c HS | 1571 | 1487 | 1372 | 603 | 1625 (*v*(C=C)_vinyl_) | - | ^6^ |
| Hb M Saskatoon | | 6c HS | 1563 | 1476 | 1367 | 578 | 1607 (*v*_10_) and 1620 (*v*(C=C)_vinyl_) | - | ^6,7^ |
| *Chlamydomonas* Hb | | 6c HS | 1558 | 1477 | 1370 | 502 | - | - | ^8^ |

Coordination number, spin state, and spectral features for various hemoproteins by resonance Raman spectroscopy.

**Table S4.** Pairs of donor/acceptor CR domains tested for hemin partitioning by mass spectrometry

| **Transfer** | **Donor** | **Acceptor** | **ratio (donor:acceptor)** | **K_rel_ (donor/acceptor)** | **K_rel_ inverse** |
| --- | --- | --- | --- | --- | --- |
| HtaA^CR2^ → ChtA^CR^ | HtaA^CR2^ | ChtA^CR^ | 3:1 | 0.03 | 32.96 |
| HtaA^CR2^ → ChtA^CR^ | HtaA^CR2^ | ChtA^CR^ | 2:1 | 0.03 | 36.21 |
| HtaA^CR2^ →ChtA^CR^ | HtaA^CR2^ | ChtA^CR^ | 1:1 | 0.05 | 18.29 |
| HtaA^CR2^ →ChtA^CR^ | HtaA^CR2^ | ChtA^CR^ | 2:1 | 0.06 | 18.06 |
| HtaA^CR2^ →ChtA^CR^ | HtaA^CR2^ | ChtA^CR^ | 1:3 | 0.05 | 19.27 |
| **AVERAGE** | | | | **0.04 ± 0.01** | **24.96 ± 8.87** |
|  |  |  |  |  |  |
| **Transfer** | **Donor** | **Acceptor** | **ratio donor:acceptor** | **K_rel_ (donor/acceptor)** | **K_rel_ inverse** |
| HtaA^CR2^ →HtaB^CR^ | HtaA^CR2^ | HtaB^CR^ | 1:1 | 3.92 | 0.26 |
| HtaA^CR2^ →HtaB^CR^ | HtaA^CR2^ | HtaB^CR^ | 3:1 | 5.56 | 0.18 |
| HtaA^CR2^ →HtaB^CR^ | HtaA^CR2^ | HtaB^CR^ | 1:3 | 5.31 | 0.19 |
| **AVERAGE** | | | | **4.93 ± 0.88** | **0.21 ± 0.04** |
|  |  |  |  |  |  |
| **Transfer** | **Donor** | **Acceptor** | **ratio donor:acceptor** | **K_rel_ (donor/acceptor)** | **K_rel_ inverse** |
| HtaA^CR1^ →HtaB^CR^ | HtaA^CR1^ | HtaB^CR^ | 1:1 | 7.51 | 0.13 |
| HtaA^CR1^ →HtaB^CR^ | HtaA^CR1^ | HtaB^CR^ | 1:1 | 4.09 | 0.24 |
| HtaA^CR1^ →HtaB^CR^ | HtaA^CR1^ | HtaB^CR^ | 1:1 | 6.88 | 0.15 |
| HtaA^CR1^ →HtaB^CR^ | HtaA^CR1^ | HtaB^CR^ | 1:3 | 4.46 | 0.22 |
| HtaA^CR1^ →HtaB^CR^ | HtaA^CR1^ | HtaB^CR^ | 1:3 | 4.42 | 0.23 |
| HtaA^CR1^ →HtaB^CR^ | HtaA^CR1^ | HtaB^CR^ | 3:1 | 6.16 | 0.16 |
| HtaA^CR1^ →HtaB^CR^ | HtaA^CR1^ | HtaB^CR^ | 3:1 | 6.76 | 0.15 |
| **AVERAGE** | | | | **5.75 ± 1.4** | **0.18 ± 0.05** |
|  |  |  |  |  |  |
| **Transfer** | **Donor** | **Acceptor** | **ratio donor:acceptor** | **K_rel_ (donor/acceptor)** | **K_rel_ inverse** |
| HtaB^CR^ →HtaA^CR2^ | HtaB^CR^ | HtaA^CR2^ | 1:1 | 0.17 | 5.8 |
| HtaB^CR^ →HtaA^CR2^ | HtaB^CR^ | HtaA^CR2^ | 3:1 | 0.19 | 5.28 |
| HtaB^CR^ →HtaA^CR2^ | HtaB^CR^ | HtaA^CR2^ | 1:3 | 0.17 | 5.9 |
| **AVERAGE** | | | | **0.18 ± 0.01** | **5.66 ± 0.33** |
|  |  |  |  |  |  |
| **Transfer** | **Donor** | **Acceptor** | **ratio donor:acceptor** | **K_rel_ (donor/acceptor)** | **K_rel_ inverse** |
| HtaA^CR1^ →HtaA^CR2^ | HtaA^CR2^ | HtaA^CR1^ | 1:1 | 0.78 | 1.28 |
| HtaA^CR1^ →HtaA^CR2^ | HtaA^CR2^ | HtaA^CR1^ | 1:3 | 0.98 | 1.02 |
| HtaA^CR1^ →HtaA^CR2^ | HtaA^CR2^ | HtaA^CR1^ | 3:1 | 0.77 | 1.29 |
| **AVERAGE** | | | | **0.84 ± 0.12** | **1.2 ± 0.15** |

Table of all CR pairs and stoichiometric ratios tested during the native ESI-MS heme equilibrium partitioning experiments. Apo or holo CR domains were mixed at various ratios (most commonly 1:3, 1:1, and 3:1, corresponding to 5:15 µM, 10:10 µM, or 15:5 µM, respectively). Mixtures were allowed to equilibrate for at least 6 hours, and a short 2-4 hour time point was also collected to ensure the equilibrium ratios were comparable and the reaction was complete.

**Table S5.** Primer sequences used in cloning of CR domain constructs.

| PCR target | primer direction | primer sequence (5' to 3') |
| --- | --- | --- |
| *htaA* (for HtaA^CR1^, aa 36-221) | forward | CACAGAGAACAGATTGGTGGATCGTTTAACTGGGGAATTCGTCAAAGTTATCG |
|  | reverse | GGTGGTGGTGCTCGAGTTAGCCGTCGAGAGCAACG |
| *htaA* (for HtaA^CR2^, aa 344-506) | forward | CACAGAGAACAGATTGGTGGAGGTGTTACTCAGGCTCACG |
|  | reverse | GGTGGTGGTGCTCGAGTTAGCCGAGTTGGGCATCGAAAG |
| *htaB* (for HtaB^CR^, aa 30-273) | forward | CACAGAGAACAGATTGGTGGAGCCGCTTCTCAGTGTGAGA |
|  | reverse | GGTGGTGGTGCTCGAGTTAGCTCCCTGACTGACTGTGC |
| *chtA* (for ChtA^CR^, aa 112-291) | forward | CACAGAGAACAGATTGGTGGAAAGAGTACTGGTGAAGCTTTGAC |
|  | reverse | GGTGGTGGTGCTCGAGTTAGCCAAGTTGTGATTTTTTGAAACTAAAC |
| pSUMO vector | forward | TAACTCGAGCACCACCACCAC |
|  | reverse | TCCACCAATCTGTTCTCTGTGAGC |

**Supplementary References**

(1) Mahoney, B. J.; Lyman, L. R.; Ford, J.; Soule, J.; Cheung, N. A.; Goring, A. K.; Ellis-Guardiola, K.; Collazo, M. J.; Cascio, D.; Ton-That, H.; Schmitt, M. P.; Clubb, R. T. Molecular Basis of Hemoglobin Binding and Heme Removal in Corynebacterium Diphtheriae. *Proc Natl Acad Sci U S A* **2025**, *122* (1), e2411833122. https://doi.org/10.1073/pnas.2411833122.

(2) Mokry, D. Z.; Nadia-Albete, A.; Johnson, M. K.; Lukat-Rodgers, G. S.; Rodgers, K. R.; Lanzilotta, W. N. Spectroscopic Evidence for a 5-Coordinate Oxygenic Ligated High Spin Ferric Heme Moiety in the *Neisseria Meningitidis* Hemoglobin Binding Receptor. *Biochimica et Biophysica Acta (BBA) - General Subjects* **2014**, *1840* (10), 3058–3066. https://doi.org/10.1016/j.bbagen.2014.06.009.

(3) Eakanunkul, S.; Lukat-Rodgers, G. S.; Sumithran, S.; Ghosh, A.; Rodgers, K. R.; Dawson, J. H.; Wilks, A. Characterization of the Periplasmic Heme-Binding Protein Shut from the Heme Uptake System of Shigella Dysenteriae. *Biochemistry* **2005**, *44* (39), 13179–13191. https://doi.org/10.1021/bi050422r.

(4) Liu, Y.; Moënne-Loccoz, P.; Hildebrand, D. P.; Wilks, A.; Loehr, T. M.; Mauk, A. G.; Ortiz de Montellano, P. R. Replacement of the Proximal Histidine Iron Ligand by a Cysteine or Tyrosine Converts Heme Oxygenase to an Oxidase. *Biochemistry* **1999**, *38* (12), 3733–3743. https://doi.org/10.1021/bi982707s.

(5) Adachi, S.; Nagano, S.; Ishimori, K.; Watanabe, Y.; Morishima, I.; Egawa, T.; Kitagawa, T.; Makino, R. Roles of Proximal Ligand in Heme Proteins: Replacement of Proximal Histidine of Human Myoglobin with Cysteine and Tyrosine by Site-Directed Mutagenesis as Models for P-450, Chloroperoxidase, and Catalase. *Biochemistry* **1993**, *32* (1), 241–252. https://doi.org/10.1021/bi00052a031.

(6) Jin, Y.; Nagai, M.; Nagai, Y.; Nagatomo, S.; Kitagawa, T. Heme Structures of Five Variants of Hemoglobin M Probed by Resonance Raman Spectroscopy. *Biochemistry* **2004**, *43* (26), 8517–8527. https://doi.org/10.1021/bi036170g.

(7) Nagai, M.; Yoneyama, Y.; Kitagawa, T. Characteristics in Tyrosine Coordinations of Four Hemoglobins M Probed by Resonance Raman Spectroscopy. *Biochemistry* **1989**, *28* (6), 2418–2422. https://doi.org/10.1021/bi00432a012.

(8) Das, T. K.; Couture, M.; Lee, H. C.; Peisach, J.; Rousseau, D. L.; Wittenberg, B. A.; Wittenberg, J. B.; Guertin, M. Identification of the Ligands to the Ferric Heme of Chlamydomonas Chloroplast Hemoglobin: Evidence for Ligation of Tyrosine-63 (B10) to the Heme. *Biochemistry* **1999**, *38* (46), 15360–15368. https://doi.org/10.1021/bi991237e.
